# Composite dietary antioxidant index is inversely and nonlinearly associated with cardiovascular disease, atherosclerotic cardiovascular disease, and cardiovascular mortality in people with dyslipidemia: evidence from NHANES 2001–2018

**DOI:** 10.3389/fnut.2024.1478825

**Published:** 2025-01-07

**Authors:** Yan Jiang, Yingying Shen

**Affiliations:** ^1^Department of Cardiovascular Disease Treatment Center, Taihe Hospital, Hubei University of Medicine, Shiyan, China; ^2^Department of Critical Care Medicine, Taihe Hospital, Hubei University of Medicine, Shiyan, China

**Keywords:** composite dietary antioxidant index, dyslipidemia, cardiovascular disease, atherosclerotic cardiovascular disease, cardiovascular mortality

## Abstract

**Background:**

Dyslipidemia is a major risk factor for cardiovascular disease (CVD) and atherosclerotic CVD (ASCVD). The composite dietary antioxidant index (CDAI), an emerging measure of combined dietary antioxidant exposure, may provide insights into the relationship between diet and CVD/ASCVD outcomes. We aimed to explore the association between CDAI and the prevalence of CVD/ASCVD, as well as CVD mortality in individuals with dyslipidemia.

**Methods:**

CDAI was assessed by integrating dietary vitamins A, C, E, zinc, selenium, and carotenoids. Dyslipidemia was diagnosed according to widely established criteria. Data on CVD/ASCVD were obtained through self-reports, while CVD mortality was obtained through prospective matching participant records with the National Death Index database. Multivariate logistic regression analysis and Cox proportional hazards regression analysis were used to explore these associations and to calculate odds ratios [OR], hazard ratios [HR], and 95% confidence intervals [CI], respectively.

**Results:**

A total of 23,126 participants with dyslipidemia from NHANES 2001–2018 were included. After adjusting for potential confounders, CDAI was inversely associated with the prevalence of both CVD and ASCVD in dyslipidemia populations (OR and 95% CI 0.979 (0.964, 0.995) and 0.977 (0.961, 0.993), respectively). Similar associations were observed between CDAI and specific types of CVD. CDAI was also inversely associated with CVD mortality in dyslipidemia participants (HR = 0.957, 95% CI = 0.939–0.976, *p* < 0.0001). Restricted cubic spline and threshold effects analyses indicated that CDAI was nonlinearly associated with CVD/ASCVD, with significant associations occurring only when CDAI≤0; however, the association of CDAI with CVD mortality was observed only when CDAI > −2. Furthermore, age, sex, and drinking were found to modify the association of CDAI with CVD/ASCVD, while body mass index influenced the relationship between CDAI and CVD mortality.

**Conclusion:**

CDAI was inversely and nonlinearly associated with both CVD/ASCVD events and CVD mortality in dyslipidemic populations. These findings highlight the potential of antioxidant dietary patterns to alleviate the CVD burden in these populations and underscore the importance of personalized strategies.

## 1 Introduction

Dyslipidemia (hyperlipidemia) is a metabolic disorder characterized by an altered blood lipid composition, specifically an increase in one or more atherogenic lipid components (e.g., hypertriglyceridemia) or a decrease in beneficial lipoproteins (e.g., high-density lipoprotein cholesterol [HDL-C]) (1, 2). The global burden of dyslipidemia has increased over the past three decades (3). Dyslipidemia is a major modifiable risk factor for cardiovascular disease (CVD), particularly atherosclerotic CVD (ASCVD). A large body of high-quality prospective clinical evidence has demonstrated that elevated serum triglycerides (TG) and low-density lipoprotein cholesterol (LDL-C) are significant risk enhancers for ASCVD, and low HDL-C has also been shown to be strongly associated with an increased risk of ASCVD (4–6). CVD remains a major cause of morbidity and mortality worldwide. In 2019, there were 523 million prevalent cases and 18.6 million deaths from CVD, reflecting a continuous increase over the past three decades (7).

Lifestyle modifications, including maintaining good dietary patterns, remain the cornerstone of non-pharmacologic prevention of CVD (8). Oxidative stress is an important pathophysiological mechanism in the development and progression of CVD (9). Notably, accumulating preclinical and clinical evidence suggests an important role for dietary antioxidants in CVD prevention. However, studies of individual dietary antioxidants have yielded inconsistent results. A large meta-analysis, including 69 prospective cohort studies, revealed that dietary vitamin C, carotenoids, and alpha tocopherol were associated with a reduced risk of CVD in the general population, whereas dietary vitamin E was not (10). Another recent retrospective cohort study showed that dietary vitamin E intake was negatively associated with the incidence of CVD in Iranian adults, whereas dietary vitamins A, C, and zinc were not (11). Similar inconsistent results were found in the association between individual dietary antioxidants and CVD mortality (12). These inconsistent studies may be partly due to the fact that assessing an individual’s dietary antioxidant intake may not accurately represent overall antioxidant exposure, thereby misestimating the effects of dietary antioxidants. In addition, no studies have demonstrated the association of dietary antioxidants with CVD prevalence and CVD mortality in people with dyslipidemia.

To address this limitation, the composite dietary antioxidant index (CDAI) was first proposed by Wright et al. to comprehensively assess dietary antioxidant exposure (13). The CDAI consists of six major dietary antioxidants, including vitamins A, C, E, zinc, selenium, and carotenoids, that represent an individual’s overall antioxidant exposure (14, 15). Recently, numerous observational clinical studies have shown that maintaining a higher CDAI is associated with a lower prevalence of CVD/ASCVD in the general population (16–18). In addition, some cohort studies have shown that CDAI is negatively associated with CVD mortality in the general or specific populations, although inconsistent findings exist (14, 19–21). However, the association of CDAI with CVD/ASCVD prevalence and CVD mortality in dyslipidemic populations remains unexplored. Addressing this knowledge gap could help reveal the preventive value of adherence to antioxidant dietary patterns for CVD morbidity and mortality in people with dyslipidemia and provide new insights for the public health community and the development of guidelines.

In this study, we utilized nationally representative data from the National Health and Nutrition Examination Survey (NHANES) to explore the independent association of the CDAI with the prevalence of CVD and its associated mortality in people with dyslipidemia. We explored the association of CDAI with CVD prevalence and mortality in participants with dyslipidemia through both a cross-sectional analysis and a prospective cohort study, respectively.

## 2 Methods

### 2.1 Study design and population

**Figure 1:**
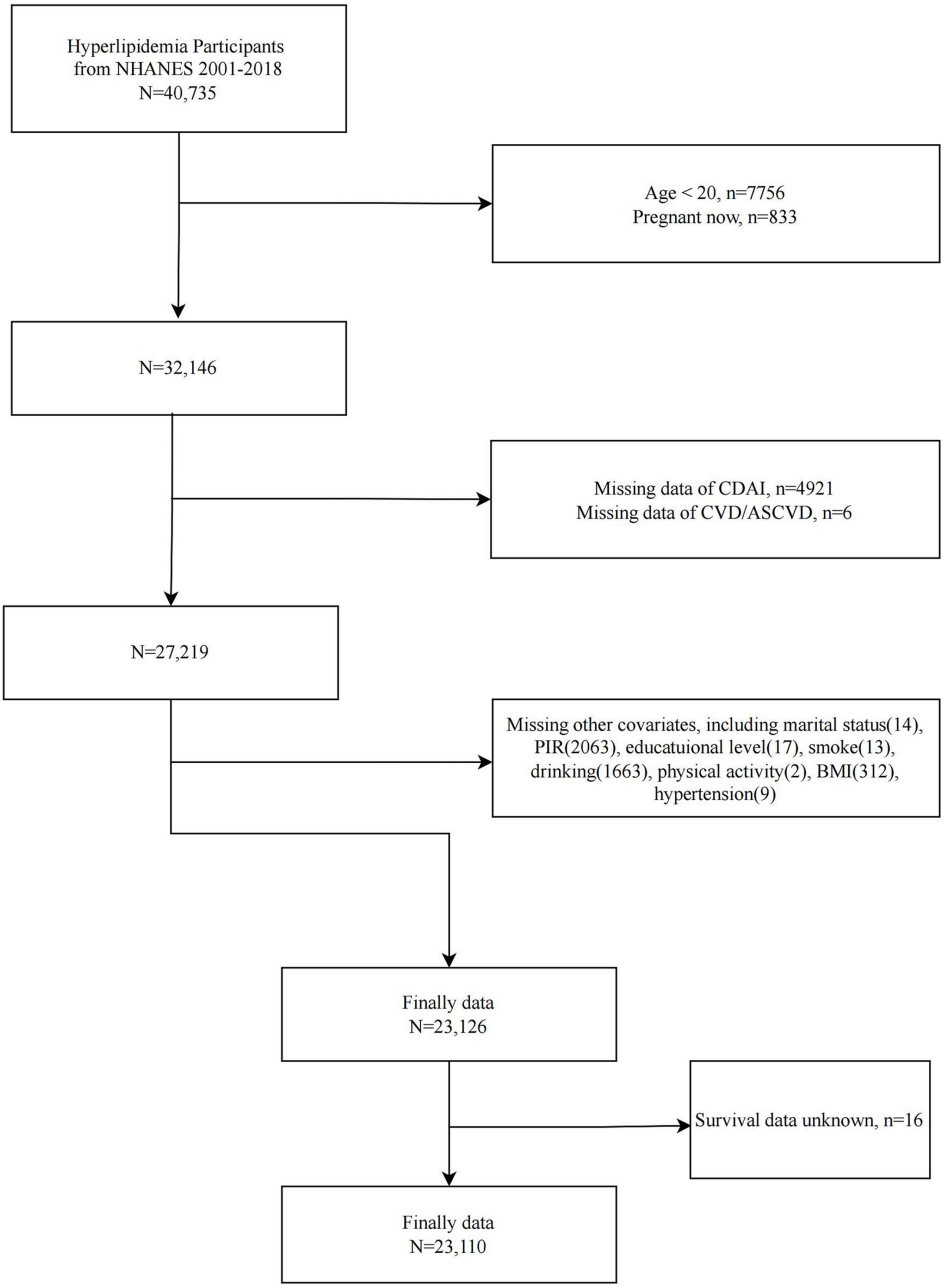
Flowchart of study population selection, NHANES 2001–2018.

NHANES is the primary epidemiologic program under the responsibility of the National Center for Health Statistics (NCHS), assessing the health and nutritional status of the U.S. ambulatory population. Since 1999, NHANES has collected relevant data continuously in two-year cycles. NHANES is a series of national, population-based, cross-sectional studies characterized by a complex, stratified, multistage probability cluster sampling design. The survey protocols for all NHANES waves were approved by the NCHS Ethics Review Board, and all participants provided written informed consent. We included 40,735 participants with dyslipidemia from nine consecutive cycles of NHANES (2001–2018). We then sequentially excluded participants <20 years of age (*n* = 7,756), pregnant (*n* = 833), missing CDAI data (*n* = 4,921), missing CVD/ASCVD information (*n* = 6), and missing covariates (*n* = 4,083). A total of 23,126 participants with dyslipidemia were included in the analysis of the between CDAI and CVD/ASCVD. In addition, after excluding participants with missing survival information, 23,110 participants were included in the analysis of the association between CDAI and CVD mortality (Figure 1).

### 2.2 Assessment of CDAI

The CDAI was assessed based on a composite of the intakes of six common dietary antioxidant micronutrients, including dietary vitamins A, C, E, zinc, selenium, and carotenoids. These dietary antioxidant intakes were calculated based on the average of two 24-h dietary recall intakes from NHANES (the first by face-to-face interview and the second by telephone 3 to 10 days later). Information on these dietary micronutrients was obtained from the NHANES dietary interview questionnaire (“What We Eat in America”), which was collected 24 h prior to the interview. Interview data files were sent electronically from the field and imported into Survey Net, and specific nutrient intakes were calculated using the U.S. Department of Agriculture’s Food and Nutrition Database for Dietary Studies (FNDDS). Based on previous widely validated methods (14, 15), we subtracted the population mean intake value from the specific intake of each dietary antioxidant, then standardized it based on the standard deviation of intake, and finally summed the scores of all six components to obtain the CDAI. The specific formula for calculating the CDAI is as follows:


$$\mathrm{CDAI}=\overset{6}{\underset{n=1}{\sum }}\frac{\mathrm{Individual}\;\mathrm{intake}-Me\mathrm{an}\;}{\mathrm{Standard}\;\mathrm{deviation}}$$

### 2.3 Evaluation of dyslipidemia

We assessed hyperlipidemia according to the National Cholesterol Education Program Adult Treatment Panel III (NCEP-ATP III) criteria and previous NHANES-related studies. Dyslipidemia was indicated by meeting one of the following five criteria: serum total cholesterol (TC) ≥200 mg/dL, TG ≥150 mg/dL, LDL-C ≥ 130 mg/dL, HDL-C ≤ 40 mg/dL (males)/≤50 mg/dL (females), or self-reported cholesterol-lowering medication use (22, 23).

### 2.4 Study outcomes determination

Our study outcomes included CVD/ASCVD prevalence and CVD mortality in people with dyslipidemia. Specific CVD types were assessed based on participants’ self-report on the corresponding Medical Conditions Questionnaire in NHANES. The presence of coronary heart disease (CHD), congestive heart failure (CHF), angina, stroke, or a heart attack (also called myocardial infarction) indicated the presence of CVD. ASCVD was defined as participants having at least one of CHD, angina, stroke, or heart attack (16). In this study, we explored the association of CDAI with CVD and specific CVD types in people with dyslipidemia. We followed the NHANES 2001–2018 dyslipidemia population until CVD-related deaths occurred or until December 31, 2019. CVD mortality information was obtained by prospectively matching the baseline dyslipidemia population to the National Death Index database. We obtained CVD mortality data through ICD-10 codes corresponding to heart disease or cerebrovascular disease, including I00-I09, I11, I13, I20-I51, and I60-I69.

### 2.5 Covariates

We included a range of covariates based on previous research, including age, gender, race/ethnicity, education level, income-poverty ratio (PIR), marital status, smoking, alcohol use, physical activity, body mass index (BMI), diabetes, and hypertension (16). Smoking was categorized as never smokers (<100 lifetime cigarettes), former smokers (≥100 lifetime cigarettes but no current cigarettes at all), or current smokers (≥100 lifetime cigarettes and currently smoking every day or someday) according to NHANES smoking-related questionnaires and previous studies (24). Drinking was categorized according to self-reports on the Alcohol Use Questionnaire and previous studies as never drinkers (<12 drinks in a lifetime), former drinkers (≥ 12 drinks in a lifetime but abstained in the last year), or current light/moderate/heavy drinkers (categorized according to sex-specific daily alcohol consumption) (25). Physical activity was categorized as no, moderate, or vigorous participation according to the NHANES Global Physical Activity Questionnaire (GPAQ) (26). BMI was assessed based on weight (kg) divided by the square of height (m) as determined by the mobile examination center. Diabetes was assessed according to self-reports, fasting blood glucose ≥7.0 mmoL/L, 2 h oral glucose tolerance test blood glucose ≥11.1 mmoL/L, glycosylated hemoglobin A1c ≥6.5%, or taking antidiabetic medication (27). Hypertension was diagnosed based on a history of hypertension, a blood pressure test ≥140/90 mmHg, or taking anti-hypertensive medications (28). DAG diagram are used to preliminarily describe their relationships (Supplementary Figure S1).

### 2.6 Statistical analysis

All analyses were appropriately weighted according to NHANES analytic guidelines to account for conditions such as nonresponse, oversampling and to obtain national estimates. Data were analyzed using R (version 4.2.3), and two-tailed *p*-values less than 0.05 were reported as statistically significant. We performed baseline analysis according to quartiles of the CDAI in people with dyslipidemia. Continuous variables were expressed as mean ± standard error and tested by weighted analysis of variance (ANOVA), while categorical variables were expressed as number (percentage) and tested by weighted chi-square analysis. To explore the association of CDAI with CVD/ASCVD and its specific types in people with dyslipidemia, we constructed multiple multivariate logistic regression models and calculated the odds ratios (OR) and 95% confidence intervals (CI). Crude models did not adjust for any covariates; model 1 adjusted for age, sex, race/ethnicity, education, PIR, and marital status; and model 2 additionally adjusted for smoking, alcohol consumption, physical activity, BMI, diabetes, and hypertension based on model 1. Kaplan–Meier (KM) survival analysis and log-rank test were used to explore the effect of different CDAI levels on CVD survival in dyslipidemia populations. Multiple multivariate Cox proportional hazards regression models were constructed to explore the effect of CDAI on CVD mortality in people with dyslipidemia and to calculated the hazard ratio (HR) and 95% CI. The crude model did not adjust for any covariates; model 1 adjusted for age, sex, race/ethnicity, education, PIR, and marital status; and model 2 additionally adjusted for smoking, alcohol consumption, physical activity, BMI, diabetes, and hypertension on top of model 1. The restricted cubic spline (RCS) model was used to explore potential nonlinear correlations and to select appropriate knots (number of knots =4; based on the lowest value of the Akaike information criterion [AIC]) for smooth curve fitting. Stratified analyses were used to explore whether the association of CDAI with CVD prevalence and CVD mortality in people with dyslipidemia remained stable across subgroups and to investigate potential effect modifiers through interaction analyses. Finally, we performed multiple sensitivity analyses. First, we excluded people with dyslipidemia who had a follow-up length less than 2 years for sensitivity analyses to exclude the effect of short follow-up length and to validate the robustness of the association of the CDAI with CVD mortality in people with dyslipidemia. Considering that diet quality may differ across populations with different levels of CDAI, we explored whether diet quality as assessed by the Healthy Eating Index-2015 (HEI-2015) (29) differed across CDAI quartiles and additionally adjusted the HEI-2015 in the fully adjusted model. Given that BMI may be an important confounder, we further explored the stability of the associations between BMI subgroups (<30 and ≥ 30) and elaborated on the differences in these associations before and after adjusting for BMI. We also explored the association of CDAI with CVD/ASCVD and CVD mortality in people without dyslipidemia to determine whether the beneficial effects of CDAI exist only in dyslipidemia populations. Finally, because dietary energy and fat intake are recognized confounders related to both CDAI and ASCVD/CVD risk, we controlled for these variables in the fully adjusted model.

## 3 Results

### 3.1 Baseline characteristics

A total of 23,126 participants with dyslipidemia were enrolled, with a mean age of 50.473 years, 48.260% male, and a mean CDAI of 0.782. As CDAI quartiles increased, participants tended to be younger, had a higher PIR, and were more likely to be male, non-Hispanic White, non-single, greater than a high school education, never/former smokers, light drinkers, and free of diabetes and hypertension. Notably, those with a higher CDAI had a lower prevalence of CVD, ASCVD, and all five CVD subtypes (Table 1).

**Table 1:** Baseline analysis of people with dyslipidemia according to CDAI quartiles, NHANES 2001–2018.

	Total (*n* = 23,126)	Q1 (*n* = 5,787)	Q2 (*n* = 5,776)	Q3 (*n* = 5,781)	Q4 (*n* = 5,782)	*p* value
CDAI	0.782 ± 0.058	−3.712 ± 0.022	−1.194 ± 0.011	1.013 ± 0.012	5.755 ± 0.082	<0.0001
Age, year	50.473 ± 0.205	50.441 ± 0.288	51.034 ± 0.264	50.670 ± 0.327	49.826 ± 0.321	0.005
PIR	3.105 ± 0.031	2.589 ± 0.039	3.021 ± 0.032	3.270 ± 0.038	3.422 ± 0.039	<0.0001
BMI	29.843 ± 0.080	29.741 ± 0.121	30.084 ± 0.124	29.841 ± 0.128	29.714 ± 0.146	0.133
Sex						<0.0001
Male	11,259 (48.260)	2,588 (41.578)	2,847 (48.709)	2,891 (49.214)	2,933 (52.146)	
Female	11,867 (51.740)	3,199 (58.422)	2,929 (51.291)	2,890 (50.786)	2,849 (47.854)	
Race/ethnicity						<0.0001
Mexican American	3,821 (7.365)	973 (7.515)	1,013 (7.965)	918 (7.075)	917 (7.003)	
Non-Hispanic Black	4,188 (8.943)	1,295 (12.187)	1,042 (9.235)	933 (7.801)	918 (7.258)	
Non-Hispanic White	11,540 (73.395)	2,643 (68.998)	2,869 (73.191)	3,005 (74.669)	3,023 (75.773)	
Other Hispanic	1827 (4.631)	520 (6.028)	446 (4.107)	462 (4.761)	399 (3.879)	
Other Race	1750 (5.666)	356 (5.272)	406 (5.503)	463 (5.693)	525 (6.087)	
Marital status						<0.0001
Non-single	14,576 (67.480)	3,340 (61.192)	3,647 (67.734)	3,795 (69.929)	3,794 (69.816)	
Single	8,550 (32.520)	2,447 (38.808)	2,129 (32.266)	1986 (30.071)	1988 (30.184)	
Education						<0.0001
< High school	2,486 (5.034)	962 (8.261)	653 (5.474)	501 (4.195)	370 (2.948)	
High school	8,817 (35.287)	2,589 (45.568)	2,293 (37.332)	2068 (32.757)	1867 (27.947)	
> High school	11,823 (59.678)	2,236 (46.171)	2,830 (57.194)	3,212 (63.049)	3,545 (69.106)	
Smoking						<0.0001
Never	11,984 (52.080)	2,678 (44.898)	2,983 (51.872)	3,083 (53.376)	3,240 (56.600)	
Former	6,472 (27.404)	1,527 (24.007)	1,649 (27.063)	1,672 (29.047)	1,624 (28.779)	
Now	4,670 (20.516)	1,582 (31.095)	1,144 (21.065)	1,026 (17.577)	918 (14.621)	
Drinking						<0.0001
Never	3,200 (10.911)	990 (13.963)	778 (10.969)	724 (10.332)	708(9.045)	
Former	4,539 (16.330)	1,354 (19.961)	1,218 (17.314)	1,018 (14.585)	949 (14.315)	
Mild	8,020 (37.908)	1,650 (29.425)	2009 (37.915)	2,169 (40.283)	2,192 (42.232)	
Moderate	3,305 (16.410)	774 (16.330)	763 (15.215)	886 (17.141)	882 (16.814)	
Heavy	4,062 (18.440)	1,019 (20.321)	1,008 (18.588)	984 (17.659)	1,051 (17.594)	
Diabetes						<0.0001
No	18,172 (83.641)	4,369 (82.578)	4,461 (81.511)	4,630 (84.919)	4,712 (85.099)	
Yes	4,954 (16.359)	1,418 (17.422)	1,315 (18.489)	1,151 (15.081)	1,070 (14.901)	
Hypertension						0.002
No	11,897 (56.624)	2,762 (55.180)	2,887 (54.499)	3,052 (57.192)	3,196 (59.045)	
Yes	11,229 (43.376)	3,025 (44.820)	2,889 (45.501)	2,729 (42.808)	2,586 (40.955)	
CHD						<0.0001
No	21,722 (95.055)	5,378 (94.514)	5,398 (94.655)	5,432 (95.479)	5,514 (96.395)	
Yes	1,304 (4.659)	379 (5.486)	349 (5.345)	328 (4.521)	248 (3.605)	
CHF						<0.0001
No	22,137 (96.984)	5,459 (96.228)	5,506 (96.591)	5,556 (97.353)	5,616 (98.137)	
Yes	923 (2.850)	301 (3.772)	260 (3.409)	212 (2.647)	150 (1.863)	
Heart attack						<0.0001
No	21,789 (95.534)	5,362 (94.463)	5,415 (95.074)	5,478 (95.815)	5,534 (96.818)	
Yes	1,307 (4.371)	415 (5.537)	349 (4.926)	298 (4.185)	245 (3.182)	
Stroke						<0.0001
No	22,043 (96.533)	5,401 (95.112)	5,504 (96.226)	5,547 (97.098)	5,591 (97.731)	
Yes	1,057 (3.362)	378 (4.888)	267 (3.774)	228 (2.902)	184 (2.269)	
Angina						0.004
No	22,205 (96.734)	5,527 (96.346)	5,519 (96.423)	5,552 (97.008)	5,607 (97.706)	
Yes	854 (3.075)	243 (3.654)	237 (3.577)	213 (2.992)	161 (2.294)	
CVD						<0.0001
No	19,933 (89.251)	4,792 (86.593)	4,929 (88.166)	5,035 (89.765)	5,177 (91.764)	
Yes	3,193 (10.749)	995 (13.407)	847 (11.834)	746 (10.235)	605 (8.236)	
ASCVD						<0.0001
No	20,192 (90.078)	4,867 (87.503)	5,001 (89.061)	5,095 (90.572)	5,229 (92.488)	
Yes	2,934 (9.922)	920 (12.497)	775 (10.939)	686 (9.428)	553 (7.512)	

Continuous variables were expressed as mean ± standard error and tested by weighted analysis of variance (ANOVA), and categorical variables were expressed as number (percentage) and tested by weighted chi-square analysis.

### 3.2 Association of CDAI with the prevalence of CVD/ASCVD in people with dyslipidemia

**Figure 2:**
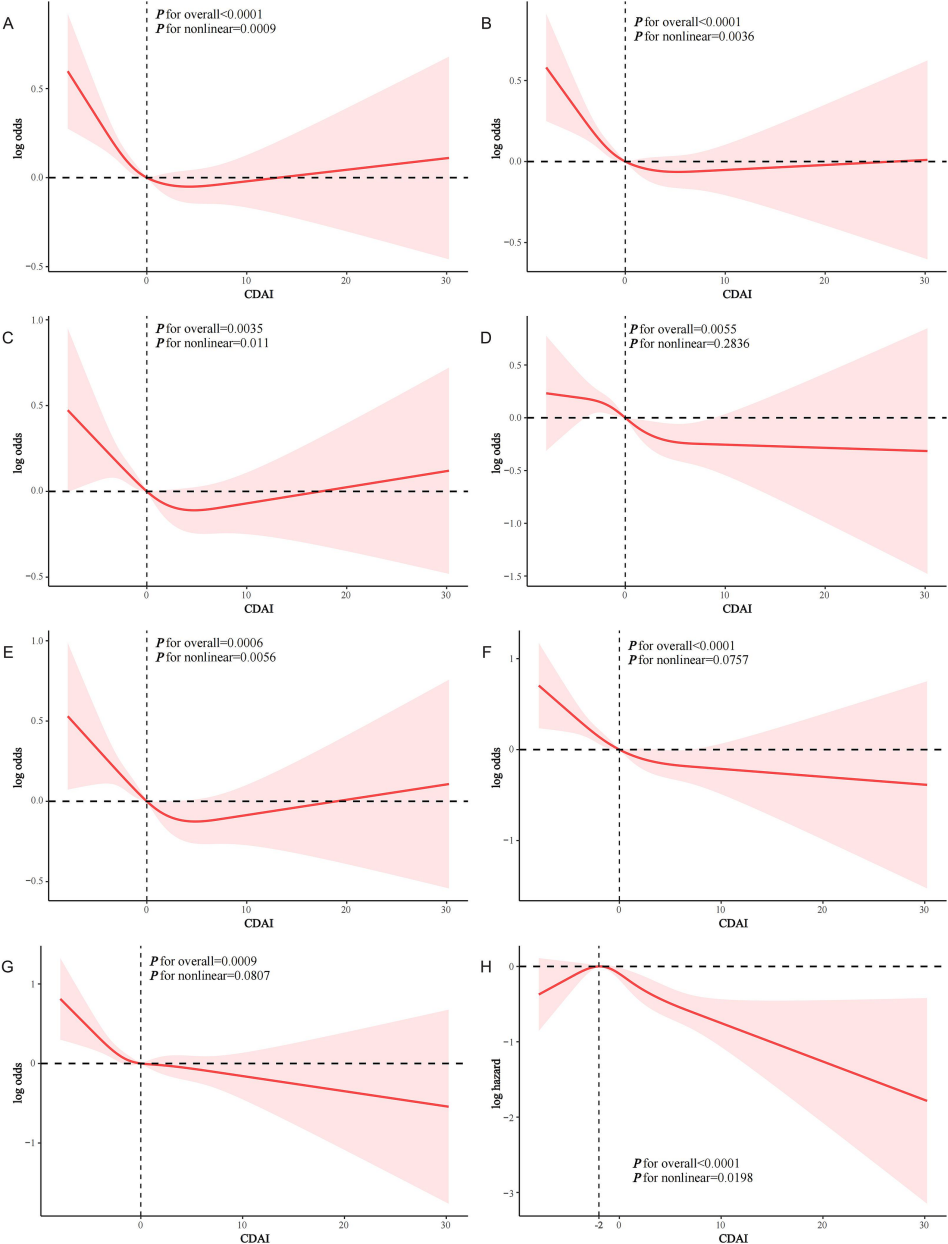
RCS analysis of the association of CDAI with CVD events in people with dyslipidemia. **(A)** CDAI and CVD; **(B)** CDAI and ASCVD; **(C)** CDAI and CHD; **(D)** CDAI and CHF; **(E)** CDAI and heart attack; **(F)** CDAI and stroke; **(G)** CDAI and angina; **(H)** CDAI and CVD mortality.

CDAI was negatively associated with the prevalence of both CVD and ASCVD in both the crude model and Model 1. In model 2, CDAI remained inversely associated with CVD (OR and 95% CI = 0.979 (0.964, 0.995), *p* = 0.0132) and ASCVD (OR and 95% CI = 0.977 (0.961, 0.993), *p* = 0.0057). Compared to Q1, participants in Q4 had significantly lower prevalence of CVD and ASCVD (OR = 0.780 and OR = 0.754, respectively; p for trend = 0.0081 and 0.0031, respectively) (Table 2). RCS analysis showed that the association of CDAI with both CVD (Figure 2A) and ASCVD (Figure 2B) in people with dyslipidemia was nonlinear (p for nonlinearity was 0.0009 and 0.0036, respectively). Threshold effect analysis revealed that the negative association of CDAI with CVD/ASCVD existed only prior to the inflection point (p for interaction was 0.0156 and 0.0219, respectively). Specifically, CDAI ≤0 was significantly associated with lower CVD and ASCVD prevalence (OR and 95% CI = 0.916 (0.866, 0.969) and 0.913 (0.861, 0.969), respectively), while no significant association was found for CDAI >0 (Table 3). Component analysis of CDAI showed that dietary vitamin A, vitamin E, and selenium intake were negatively associated with the prevalence of CVD/ASCVD in individuals with dyslipidemia, whereas vitamin C, zinc, and carotenoids were not (Supplementary Tables S1, S2).

**Table 2:** Association of CDAI with prevalence of CVD/ASCVD in people with dyslipidemia.

	Crude Model OR (95%CI) *P*-value	Model 1 OR (95%CI) *P*-value	Model 2 OR (95%CI) *P*-value
CVD			
CDAI	0.950 (0.936, 0.964) <0.0001	0.971 (0.956, 0.987) 0.0006	**0.979 (0.964, 0.995) 0.0132**
CDAI quartile			
Q1	Ref.	Ref.	Ref.
Q2	0.868 (0.764, 0.985) 0.0303	0.903 (0.780, 1.046) 0.1768	0.906 (0.779, 1.054) 0.2035
Q3	0.737 (0.636, 0.853) 0.0001	0.835 (0.706, 0.987) 0.0370	0.874 (0.739, 1.033) 0.1177
Q4	0.580 (0.503, 0.669) <0.0001	0.731 (0.616, 0.866) 0.0004	**0.780 (0.656, 0.926) 0.0054**
P for trend	<0.0001	0.0005	**0.0081**
ASCVD			
CDAI	0.949 (0.935, 0.963) <0.0001	0.969 (0.953, 0.985) 0.0003	**0.977 (0.961, 0.993) 0.0057**
CDAI quartile			
Q1	Ref.	Ref.	Ref.
Q2	0.861 (0.752, 0.986) 0.0323	0.886 (0.757, 1.037) 0.1335	0.892 (0.758, 1.050) 0.1715
Q3	0.729 (0.624, 0.853) 0.0001	0.813 (0.680, 0.972) 0.0252	0.852 (0.712, 1.021) 0.0851
Q4	0.569 (0.491, 0.659) <0.0001	0.706 (0.593, 0.840) 0.0001	**0.754 (0.634, 0.898) 0.0020**
P for trend	<0.0001	0.0002	**0.0031**

CVD, cardiovascular disease; CDAI quartile, composite dietary antioxidant index quartile; ASCVD, atherosclerotic cardiovascular disease; OR, odds ratio; 95% CI, 95% confidence interval; Crude models did not adjust for any covariates; model 1 adjusted for age, sex, race/ethnicity, education, PIR, and marital status; and model 2 additionally adjusted for smoking, alcohol consumption, physical activity, BMI, diabetes, and hypertension based on model 1.

**Table 3:** Threshold effect analysis of the association between CDAI and the prevalence of CVD/ASCVD in people with dyslipidemia.

	CDAI ≤0	CDAI >0	P-interaction
	OR (95%CI) *P*-value	OR (95%CI) *P*-value	
CVD			
CDAI	**0.916 (0.866, 0.969) 0.0029**	0.991 (0.968, 1.014) 0.4380	**0.0156**
ASCVD			
CDAI	**0.913 (0.861, 0.969) 0.0032**	0.988 (0.965, 1.011) 0.2939	**0.0219**

CVD, cardiovascular disease; ASCVD, atherosclerotic cardiovascular disease; OR, odds ratio; 95% CI, 95% confidence interval; Adjusted for age, sex, race/ethnicity, education, PIR, marital status, smoking, alcohol consumption, physical activity, BMI, diabetes, and hypertension.

### 3.3 Association of CDAI with the prevalence of specific CVD types in people with dyslipidemia

In Model 2, CDAI was inversely associated with CHD (OR = 0.978, 95% CI = 0.957–1.000, *p* = 0.0492), CHF (OR = 0.965, 95% CI = 0.940–0.991, *p* = 0.0096), heart attack (OR = 0.974, 95% CI = 0.949–0.999, *p* = 0.0447), stroke (OR = 0.957, 95% CI = 0.934–0.981, *p* = 0.0008), and angina (OR = 0.967, 95% CI = 0.937–0.997, *p* = 0.0349). A higher CDAI was significantly associated with lower odds of each specific CVD type (p for trend all <0.05) (Supplementary Table S3). RCS analysis showed a nonlinear relationship between CDAI and the prevalence of CHD (Figure 2C) and heart attack (Figure 2E) (p for nonlinearity was 0.011 and 0.0056, respectively). In contrast, CDAI was linearly associated with the odds of other types of CVD including CHF (Figure 2D), stroke (Figure 2F), and angina (Figure 2G). Threshold effect analysis showed that the negative association of CDAI with CHD and heart attack was existed only before the inflection point (CDAI ≤0) in the dyslipidemia population (Supplementary Table S4).

### 3.4 Association of CDAI with CVD mortality in people with dyslipidemia

**Figure 3:**
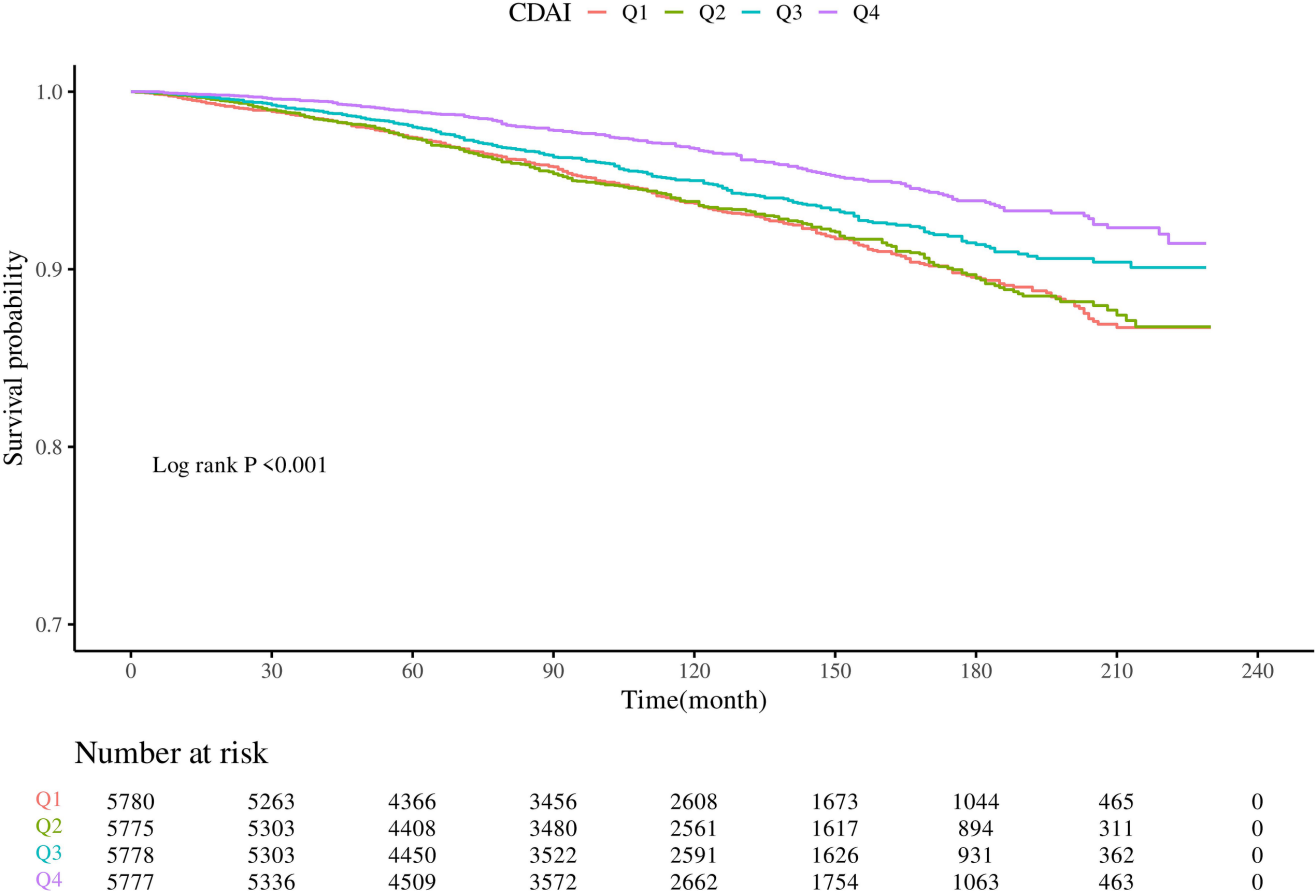
KM survival analysis of CDAI and CVD-related survival in dyslipidemia populations.

KM survival curves indicated that higher CDAI quartiles were significantly associated with increased CVD survival in the dyslipidemia population compared to Q1 (log-rank test *p* < 0.001) (Figure 3). After a median follow-up of 116 (interquartile range: 71–163) months, 1,075 CVD-related deaths were recorded. In Model 2, CDAI was inversely associated with CVD mortality (HR = 0.957, 95%CI = 0.939–0.976, *p* < 0.0001). Participants in Q4 with higher CDAI had significantly lower CVD mortality compared to Q1 (HR = 0.745; p for trend<0.001) (Table 4). RCS analysis showed that CDAI was nonlinearly associated with CVD mortality (p for nonlinearity = 0.0198) (Figure 2H). Threshold effect analysis indicated that this negative association was significant after the inflection point (HR and 95% CI = 0.924 (0.902, 0.947), *p* < 0.0001) (Table 5). Analysis of CDAI components showed that dietary intake of vitamin E, zinc, selenium, and carotenoid were significantly negatively associated with CVD mortality, whereas dietary vitamins A and C were not (Supplementary Table S5).

**Table 4:** Association of CDAI with CVD mortality in dyslipidemia population.

	Crude Model HR (95%CI) *P*-value	Model 1 HR (95%CI) *p*-value	Model 2 HR (95%CI) *p*-value
CDAI	0.933 (0.915,0.951) <0.0001	0.953 (0.936,0.971) <0.0001	**0.957 (0.939,0.976) < 0.0001**
CDAI quartile			
Q1	ref	ref	ref
Q2	1.097 (0.892,1.351) 0.38	1.215 (0.995,1.483) 0.056	1.180 (0.962,1.448) 0.112
Q3	0.788 (0.645,0.963) 0.02	0.949 (0.783,1.150) 0.595	0.959 (0.802,1.147) 0.648
Q4	0.575 (0.460,0.718) <0.0001	0.710 (0.570,0.886) 0.002	**0.745 (0.603,0.921) 0.006**
*P* for trend	<0.0001	<0.0001	**<0.001**

CDAI quartile, composite dietary antioxidant index quartile; HR, hazard ratio; 95% CI, 95% confidence interval; Crude models did not adjust for any covariates; model 1 adjusted for age, sex, race/ethnicity, education, PIR, and marital status; and model 2 additionally adjusted for smoking, alcohol consumption, physical activity, BMI, diabetes, and hypertension based on model 1.

**Table 5:** Threshold effect analysis of the association between CDAI and CVD mortality in dyslipidemia populations.

	CDAI ≤-2	CDAI >-2	*P*-interaction
	HR (95%CI) *P*-value	HR (95%CI) *P*-value	
CVD mortality			
CDAI	1.013 (0.933, 1.099) 0.7639	**0.924 (0.902, 0.947) < 0.0001**	**0.0001**

CVD mortality, cardiovascular disease mortality; HR, hazard ratio; 95% CI, 95% confidence interval; Adjusted for age, sex, race/ethnicity, education, PIR, marital status, smoking, alcohol consumption, physical activity, BMI, diabetes, and hypertension.

### 3.5 Stratified analysis

**Figure 4:**
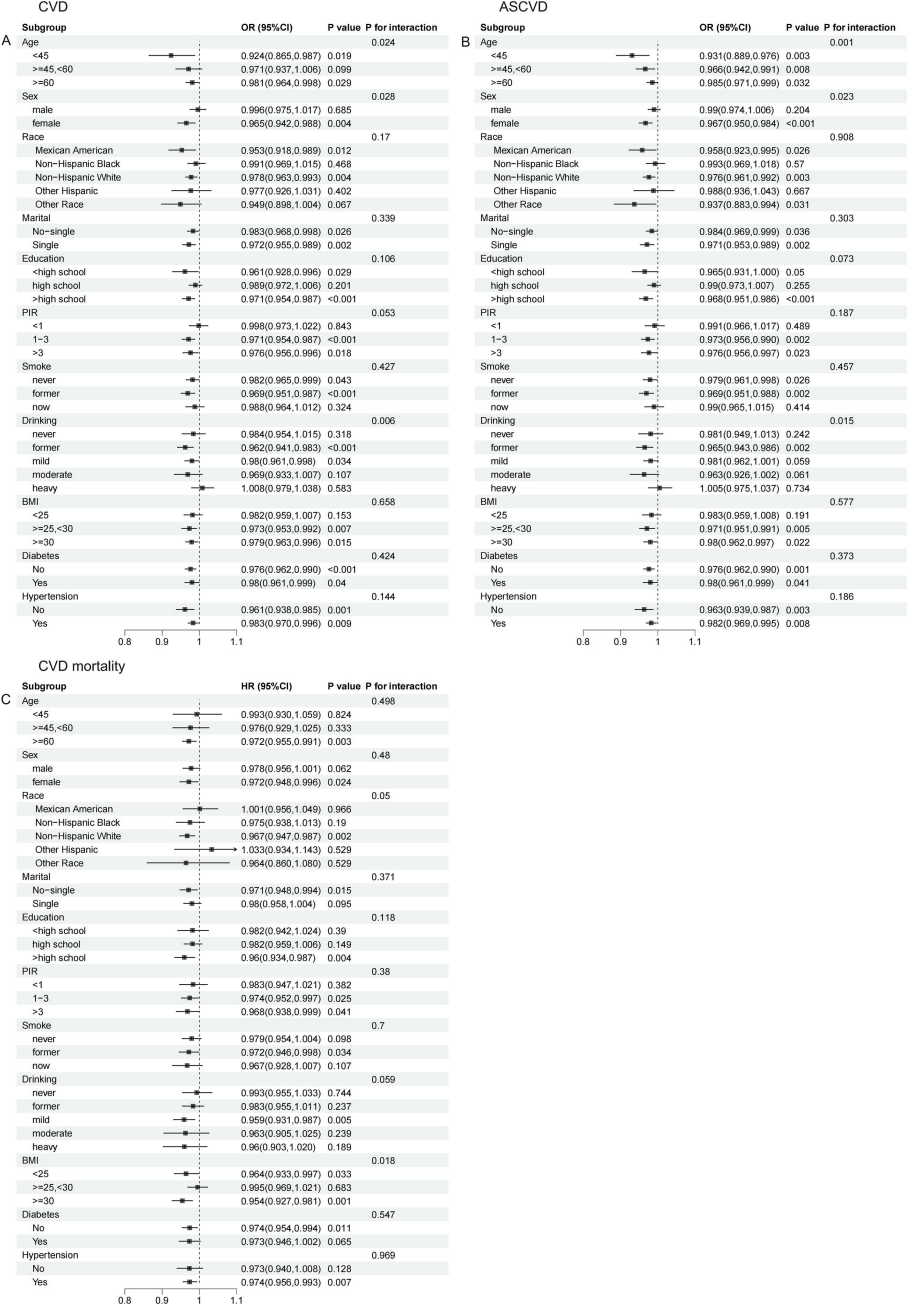
Stratified analysis of the association of CDAI with CVD/ASCVD and CVD mortality in people with dyslipidemia. **(A)** CVD; **(B)** ASCVD; **(C)** CVD mortality.

Interaction analyses showed that age, sex, and alcohol consumption all significantly influenced the association of CDAI with the prevalence of CVD (Figure 4A) and ASCVD (Figure 4B) in dyslipidemia populations (p for interaction all <0.05). These effects were particularly pronounced in those <45 years, females, and former drinkers. Additionally, BMI significantly modified the association between CDAI and CVD mortality (p for interaction = 0.018), with a stronger association observed in those with a BMI ≥30 kg/m^2^ (Figure 4C).

### 3.6 Sensitivity analysis

Excluding participants with a follow-up duration of less than 2 years did not significantly alter the results. In model 2, CDAI remained inversely associated with CVD mortality in the dyslipidemia population (HR and 95% CI = 0.960 (0.940, 0.980), *p* < 0.001), confirming the stability of the findings (Supplementary Table S6). Notably, participants in higher CDAI quartiles exhibited higher HEI-2015 scores (Supplementary Table S7). However, additional adjustment of HEI-2015 to model 2 did not significantly affect the results (CVD: OR 0.981, *p* = 0.035; ASCVD: OR 0.979, *p* = 0.018; CVD mortality: HR 0.949, *p* < 0.0001) (Supplementary Table S8). Stratified analyses by BMI (<30 and ≥ 30) similarly demonstrated that BMI was a significant effect modifier of the association between CDAI and CVD mortality in people with dyslipidemia (p for interaction 0.007), with a stronger association in those with a BMI ≥30 kg/m2 (Supplementary Table S9). In addition, we found little difference between the associations before and after adjusting for BMI in the fully adjusted model, suggesting that adjusting for BMI did not significantly affect these associations (Supplementary Tables S10, S11). In individuals without dyslipidemia, the association of CDAI with CVD/ASCVD and CVD mortality was largely absent (CVD: fully-adjusted OR 0.984, *p* = 0.108; ASCVD: fully-adjusted OR 0.970, *p* = 0.051; CVD mortality: fully-adjusted HR 1.001, *p* = 0.911) (Supplementary Tables S12–S14), suggesting that the preventive effect of CDAI on CVD outcomes may be limited to individuals with dyslipidemia. Notably, participants in higher CDAI quartiles demonstrated higher intakes of essential nutrients, including Vitamins A, C, E, Zinc, Selenium, and Carotenoids, along with increased total energy and fat intake (Supplementary Table S15). However, further adjustment for the total energy and fat intake did not significantly alter the results (CVD: OR 0.979, *p* = 0.013; ASCVD: OR 0.977, *p* = 0.006; CVD mortality: HR 0.956, *p* < 0.0001) (Supplementary Tables S16, S17).

## 4 Discussion

In this national cross-sectional analysis, CDAI was negatively and nonlinearly associated with the prevalence of both CVD and ASCVD in individuals with dyslipidemia. Notably, these associations were significant only when CDAI was ≤0. Similar negative associations were observed between CDAI and specific CVD types. In the prospective study, CDAI was also inversely and nonlinearly associated with CVD mortality in dyslipidemia populations. However, this association was only significant at CDAI > −2. Additionally, factors such as age, sex, and drinking influenced the association of CDAI with CVD/ASCVD prevalence, whereas BMI was identified as a significant modifier of the association between CDAI and CVD mortality.

To the best of our knowledge, this is the first study to explore the association of CDAI as an emerging indicator of overall dietary antioxidant exposure with CVD/ASCVD prevalence and CVD mortality in people with dyslipidemia. Previous research has consistently shown that CDAI is negatively associated with the prevalence of CVD/ASCVD in the general population. A cross-sectional analysis from NHANES (2013–2018) demonstrated that CDAI was negatively associated with ASCVD risk in postmenopausal women (17). Similarly, another NHANES study (2001–2018) reported that CDAI was negatively associated with ASCVD prevalence in the general adult population (OR = 0.968, 95% CI = 0.959–0.978, *p* < 0.001) (16). A recent cross-sectional analysis from NHANES (2011–2020) showed that CDAI was negatively and non-linearly associated with the prevalence of CVD in the general population (compared to Q1: OR = 0.71, 95% CI = 0.59–0.85, *p* < 0.001 for CDAI at Q4); however, this association was no longer significant when CDAI was treated as a continuous variable (OR = 0.98, 95% CI = 0.96–1.00, *p* = 0.052) (18). A substantial body of cross-sectional evidence suggested that CDAI was also negatively associated with the prevalence of specific CVD types in the general population. In a recent cross-sectional analysis from NHANES, Ma et al. found that CDAI was inversely associated with odds of CHD in the general adult population (OR = 0.95, 95% CI = 0.92–0.97, *p* < 0.001) and exhibited a nonlinear association (inflection point = 0.16) (30). Another NHANES study reported that CDAI was negatively and nonlinearly associated with stroke prevalence in the U.S. adult population (OR = 0.96, 95% CI = 0.94–0.98, *p* < 0.001; p for nonlinearity <0.001, inflection point = 3.66) (31). Similarly, a recent cross-sectional analysis from NHANES (2001–2018) including 29,101 participants >40 years of age revealed that the CDAI was negatively associated with the prevalence of CHF in the general population (OR = 0.96, 95% CI = 0.94–0.99, *p* = 0.02; compared to Q1, the OR for the CDAI at Q4 = 0.68, 95% CI = 0.52–0.89, *p* = 0.01) (32), consistent with another NHANES study (33). However, the association of CDAI with the prevalence of angina and heart attack is currently unexplored. Our study provides new insights by demonstrating that CDAI was significantly and inversely associated with the prevalence of CVD, ASCVD, and specific CVD types in people with dyslipidemia, suggesting for the first time that higher dietary antioxidant potential may help to reduce the prevalence of CVD/ASCVD in this population.

Of note, some studies have shown a negative correlation between CDAI and CVD mortality in general or specific populations as well, but the findings have been inconsistent. A prospective cohort study similarly from NHANES demonstrated that higher CDAI was associated with reduced CVD mortality in the general adult population (HR = 0.81, 95% CI = 0.66–0.99, *p* = 0.040 for CDAI at Q4 compared to Q1; p for trend = 0.024) and exhibited a linear correlation in RCS (20). Another prospective cohort study using NHANES (1999–2018) demonstrated that CDAI was negatively associated with CVD mortality in people with diabetes mellitus (HRs and 95% CIs for CDAI at Q3 and Q4 compared to Q1 were 0.58 (0.38–0.88) and 0.47 (0.30–0.75), respectively), showing a nonlinear relationship (19). A recent longitudinal cohort study similarly (NHANES 2001–2018) suggested that CDAI lost its association with CVD mortality in people with hypertension after adjusting for confounders (HRs and 95% CIs for being in Q2, Q3, and Q4 compared to Q1 were 0.81 (0.64, 1.01), 0.84 (0.67, 1.04), and 0.83 (0.67, 1.04), respectively; p for trend = 0.13) (21). Similarly, a recent population-based cohort study (NHANES 2001–2018) found no association between CDAI and CVD mortality in people with metabolic syndrome (HR = 0.98, 95% CI = 0.94–1.02, *p* = 0.3128 per unit increase; compared to Q1, HRs and 95% CIs for those at Q2, Q3, and Q4 were 1.04 (0.79, 1.38), 0.91 (0.66, 1.26), and 0.95 (0.65, 1.38), respectively) (14). Interestingly, a cohort study utilizing NHANES (2007–2018) demonstrated that CDAI lost its association with CVD mortality in people with diabetes after adjusting for confounders (compared to Q1, the HRs and 95% CIs for CDAI at Q2, Q3, and Q4 were 0.72 (0.44, 1.18), 1.13 (0.69, 1.85), and 0.74 (0.49, 1.12), respectively; p for trend = 0.59) (34), inconsistent with the findings of Yang et al. (19). These results suggest that the effect of CDAI on CVD mortality varies significantly across populations and may be influenced by sample size. Our study demonstrated for the first time that CDAI was negatively correlated with CVD mortality in people with dyslipidemia, suggesting that maintaining a higher CDAI has a potential preventive role for CVD mortality in people with dyslipidemia. Given that populations with dyslipidemia are at increased risk for CVD morbidity and mortality, these findings may suggest that adherence to antioxidant dietary patterns assessed by the CDAI could serve as a dietary intervention for excess CVD mortality in dyslipidemia populations.

Despite growing interest in diet as a factor in cardiovascular risk prevention for people with dyslipidemia, real-world research remains limited. A population-based study using NHANES (2001–2016) demonstrated that marine polyunsaturated fatty acids were associated with reduced odds of CVD (OR = 0.71, 95%CI = 0.55–0.91) and CVD mortality (HR = 0.68, 95%CI: 0.52–0.90) among people with dyslipidemia but had no significant impact on prevalence of CHD, CHF, and heart attack (35). The role of dietary antioxidants in the prevention of cardiovascular event risk in people with dyslipidemia remains understudied. A cohort study using NHANES (1999–2018) demonstrated that adherence to higher dietary oxidative balance score, another emerging metric for assessing overall exposure to dietary antioxidants, was negatively associated with CVD mortality in people with dyslipidemia (per unit increase HR = 0.98, 95% CI = 0.97–1.00, *p* = 0.0052) (36). While the association of individual dietary antioxidants, such as various vitamins and minerals, with the incidence of CVD and its subtypes has been extensively studied, results remain inconsistent (10–12, 37). A more comprehensive approach, measuring dietary antioxidants through CDAI and considering their interactions, can better capture the full impact of dietary antioxidants. These findings underscore the need for clinicians and public health experts to prioritize the preventive value of dietary antioxidant patterns for CVD/ASCVD and related mortality in people with dyslipidemia, and to promote the importance of adherence to antioxidant-rich diets in individuals with hyperlipidemia.

In addition to common dietary antioxidant micronutrients, plant extracts with antioxidant properties may offer potential for improving CVD outcomes. Previous studies have shown that certain plant extracts exhibit antioxidant activity and may improve SIRT1 levels in cardiomyocytes (38, 39). Accumulating evidence also suggests that plant extracts may ameliorate dyslipidemia (40). However, the impact of antioxidant plant extracts on CVD outcomes in populations with dyslipidemia remains underexplored in real-world exploration.

Our findings suggest that the preventive value of CDAI for CVD/ASCVD in dyslipidemia is population-specific, highlighting the need for individualized prevention strategies. Previous NHANES-related studies have similarly shown that the association of CDAI with the odds of CVD in the general population exists only in women (18). Another study utilizing NHANES 2001–2018 demonstrated that the association of CDAI with ASCVD in the general population was stronger in those <65 years of age (16). Gender-related disparities in hormonal, genetic, and epigenetic factors likely contribute to distinct cardiovascular risk profiles in men and women (41). Younger adults may have lower CVD/ASCVD risk and better CDAI adherence compared to older populations, and thus CDAI may have better protective value. The effects of alcohol consumption on cardiovascular risk are still under discussion (42, 43), and thus the specific mechanisms underlying the beneficial effects of CDAI in former drinkers still need to be further elucidated. In addition, although the association between BMI and CVD mortality remains controversial (44), we speculate that obesity, together with dyslipidemia, increases CVD mortality and amplifies the protective role of CDAI.

This study highlights the potential of the CDAI as a valuable tool in CVD management. Clinicians should consider incorporating CDAI into routine screenings for patients at risk of CVD, particularly those with dyslipidemia, to identify individuals who may benefit from dietary interventions. Given the nonlinear relationship between CDAI and CVD, personalized dietary strategies focusing on antioxidant-rich foods could be particularly beneficial for patients with low CDAI scores. Additionally, public health initiatives should promote antioxidant-rich diets as part of broader strategies to reduce CVD risk in high-risk populations.

Our study has several significant advantages. It was a nationally representative, population-based study with a large multiethnic sample, enhancing generalizability of the findings. We effectively controlled for confounding factors to minimize bias. CVD mortality was prospectively collected with long follow-up, ensuring reliability. However, there are limitations to our study. The association between CDAI and CVD/ASCVD prevalence was explored by cross-sectional analyses, so causal relationships could not be established, and residual confounding may have influenced the results. As a large epidemiologic survey, dietary assessments and diagnoses of all CVD types were obtained based on self-report and may be subject to recall bias. In addition, the absence of relevant data from NHANES prevented us from examining the impact of other factors such as genetic susceptibility to CVD.

## 5 Conclusion

In a national population-based study, CDAI was inversely and nonlinearly associated with the prevalence of CVD/ASCVD in populations with dyslipidemia and these associations existed only at CDAI ≤0. Similar associations were found between CDAI and specific CVD types. Similar inverse and nonlinear associations was observed between CDAI with CVD mortality in dyslipidemia populations, but only when CDAI was > − 2. Age, sex, and drinking influenced the association of CDAI with CVD/ASCVD, whereas BMI influenced the effect of CDAI on CVD mortality. Future well-designed prospective cohort studies are needed to validate these findings.

## Data Availability

Publicly available datasets were analyzed in this study. This data can be found at: This study analyzed publicly available datasets and can be found at https://www.cdc.gov/nchs/nhanes/.
