# Supplementary material for: Composite dietary antioxidant index is inversely and nonlinearly associated with cardiovascular disease, atherosclerotic cardiovascular disease, and cardiovascular mortality in people with dyslipidemia: evidence from NHANES 2001–2018
Source: Front Nutr. 2025 Jan 7;11:1478825. doi: 10.3389/fnut.2024.1478825 (PMC11753228; doi:10.3389/fnut.2024.1478825)

**Table S1**. Association of CDAI components with CVD prevalence in dyslipidemia populations.

| **CVD** | **Crude model**  **OR (95%CI) P-value** | **Model 1**  **OR (95%CI) P-value** | **Model 2**  **OR (95%CI) P-value** |
| --- | --- | --- | --- |
| **Vitamin A** | 0.9999 (0.9998, 1.0000) 0.2772 | 0.9998 (0.9996, 0.9999) 0.0063 | 0.9998 (0.9997, 1.0000) 0.0220 |
| **Vitamin A Quartile** |  |  |  |
| **Q1** | Ref. | Ref. | Ref. |
| **Q2** | 1.0171 (0.8770, 1.1796) 0.8227 | 0.8714 (0.7410, 1.0246) 0.0982 | 0.8912 (0.7536, 1.0538) 0.1807 |
| **Q3** | 1.0485 (0.9137, 1.2031) 0.5011 | 0.8140 (0.6914, 0.9585) 0.0149 | 0.8557 (0.7230, 1.0127) 0.0724 |
| **Q4** | 0.8959 (0.7842, 1.0234) 0.1078 | 0.6803 (0.5825, 0.7945) <0.0001 | 0.7378 (0.6305, 0.8635) 0.0002 |
| **p for trend** | 0.9690 (0.9312, 1.0084) 0.1241 | 0.8846 (0.8420, 0.9295) <0.0001 | 0.9091 (0.8650, 0.9553) 0.0003 |
| **Vitamin C** | 0.9997 (0.9991, 1.0003) 0.2684 | 0.9994 (0.9987, 1.0002) 0.1366 | 1.0002 (0.9995, 1.0009) 0.6065 |
| **Vitamin C Quartile** |  |  |  |
| **Q1** | Ref. | Ref. | Ref. |
| **Q2** | 1.0425 (0.9162, 1.1862) 0.5284 | 0.9459 (0.8153, 1.0974) 0.4644 | 0.9939 (0.8548, 1.1557) 0.9372 |
| **Q3** | 1.0239 (0.8948, 1.1716) 0.7321 | 0.8421 (0.7350, 0.9648) 0.0146 | 0.9346 (0.8216, 1.0632) 0.3059 |
| **Q4** | 0.9329 (0.8226, 1.0579) 0.2808 | 0.8107 (0.7006, 0.9380) 0.0056 | 0.9553 (0.8215, 1.1109) 0.5537 |
| **p for trend** | 0.9789 (0.9396, 1.0199) 0.3103 | 0.9277 (0.8873, 0.9700) 0.0012 | 0.9798 (0.9358, 1.0259) 0.3865 |
| **Vitamin E** | 0.9692 (0.9577, 0.9808) <0.0001 | 0.9794 (0.9670, 0.9921) 0.0019 | 0.9840 (0.9714, 0.9968) 0.0156 |
| **Vitamin E Quartile** |  |  |  |
| **Q1** | Ref. | Ref. | Ref. |
| **Q2** | 0.8548 (0.7553, 0.9675) 0.0142 | 0.8745 (0.7612, 1.0047) 0.0605 | 0.9021 (0.7817, 1.0411) 0.1615 |
| **Q3** | 0.7688 (0.6641, 0.8901) 0.0006 | 0.8244 (0.6970, 0.9751) 0.0259 | 0.8447 (0.7135, 1.0001) 0.0525 |
| **Q4** | 0.6563 (0.5687, 0.7574) <0.0001 | 0.7516 (0.6364, 0.8877) 0.0010 | 0.8046 (0.6759, 0.9578) 0.0160 |
| **p for trend** | 0.8721 (0.8326, 0.9135) <0.0001 | 0.9129 (0.8651, 0.9633) 0.0012 | 0.9311 (0.8799, 0.9852) 0.0146 |
| **Zinc** | 0.9830 (0.9678, 0.9984) 0.0321 | 0.9963 (0.9843, 1.0083) 0.5441 | 0.9966 (0.9857, 1.0076) 0.5442 |
| **Zinc Quartile** |  |  |  |
| **Q1** | Ref. | Ref. | Ref. |
| **Q2** | 0.8365 (0.7279, 0.9614) 0.0131 | 0.8995 (0.7693, 1.0516) 0.1863 | 0.8928 (0.7541, 1.0570) 0.1906 |
| **Q3** | 0.8100 (0.7080, 0.9268) 0.0026 | 0.9588 (0.8210, 1.1197) 0.5960 | 0.9544 (0.8076, 1.1278) 0.5848 |
| **Q4** | 0.6495 (0.5604, 0.7527) <0.0001 | 0.8111 (0.6846, 0.9610) 0.0170 | 0.8115 (0.6812, 0.9667) 0.0210 |
| **p for trend** | 0.8763 (0.8354, 0.9191) <0.0001 | 0.9459 (0.8958, 0.9989) 0.0478 | 0.9463 (0.8936, 1.0022) 0.0621 |
| **Selenium** | 0.9957 (0.9943, 0.9970) <0.0001 | 0.9984 (0.9970, 0.9998) 0.0262 | 0.9983 (0.9969, 0.9997) 0.0227 |
| **Selenium Quartile** |  |  |  |
| **Q1** | Ref. | Ref. | Ref. |
| **Q2** | 0.8959 (0.7865, 1.0205) 0.1004 | 0.9715 (0.8367, 1.1281) 0.7055 | 0.9836 (0.8440, 1.1464) 0.8330 |
| **Q3** | 0.7294 (0.6254, 0.8507) 0.0001 | 0.8600 (0.7202, 1.0269) 0.0980 | 0.8398 (0.6950, 1.0147) 0.0731 |
| **Q4** | 0.5597 (0.4752, 0.6593) <0.0001 | 0.7987 (0.6648, 0.9597) 0.0179 | 0.7836 (0.6525, 0.9411) 0.0103 |
| **p for trend** | 0.8253 (0.7826, 0.8703) <0.0001 | 0.9240 (0.8689, 0.9825) 0.0129 | 0.9154 (0.8597, 0.9747) 0.0067 |
| **Carotenoid** | 1.0000 (1.0000, 1.0000) 0.0006 | 1.0000 (1.0000, 1.0000) 0.1029 | 1.0000 (1.0000, 1.0000) 0.5419 |
| **Carotenoid Quartile** |  |  |  |
| **Q1** | Ref. | Ref. | Ref. |
| **Q2** | 0.8932 (0.7842, 1.0173) 0.0911 | 0.9538 (0.8258, 1.1017) 0.5211 | 0.9696 (0.8370, 1.1232) 0.6815 |
| **Q3** | 0.8012 (0.6832, 0.9396) 0.0072 | 0.9267 (0.7737, 1.1100) 0.4100 | 0.9746 (0.8164, 1.1635) 0.7763 |
| **Q4** | 0.7545 (0.6557, 0.8683) 0.0001 | 0.8445 (0.7185, 0.9925) 0.0424 | 0.9045 (0.7682, 1.0650) 0.2309 |
| **p for trend** | 0.9089 (0.8671, 0.9527) 0.0001 | 0.9477 (0.8987, 0.9994) 0.0496 | 0.9707 (0.9204, 1.0237) 0.2756 |

Crude models did not adjust for any covariates; model 1 adjusted for age, sex, race/ethnicity, education, PIR, and marital status; and model 2 additionally adjusted for smoking, alcohol consumption, physical activity, BMI, diabetes, and hypertension based on model 1.

**Table S2**. Association of CDAI components with ASCVD prevalence in dyslipidemia populations.

| **ASCVD** | **Crude model**  **OR (95%CI) P-value** | **Model 1**  **OR (95%CI) P-value** | **Model 2**  **OR (95%CI) P-value** |
| --- | --- | --- | --- |
| **Vitamin A** | 0.9999 (0.9998, 1.0000) 0.0482 | 0.9997 (0.9996, 0.9999) <0.0001 | 0.9998 (0.9997, 0.9999) 0.0004 |
| **Vitamin A Quartile** |  |  |  |
| **Q1** | Ref. | Ref. | Ref. |
| **Q2** | 0.9973 (0.8537, 1.1650) 0.9725 | 0.8443 (0.7142, 0.9982) 0.0497 | 0.8666 (0.7286, 1.0308) 0.1086 |
| **Q3** | 1.0348 (0.8961, 1.1951) 0.6417 | 0.7928 (0.6666, 0.9430) 0.0098 | 0.8343 (0.6983, 0.9968) 0.0483 |
| **Q4** | 0.8967 (0.7778, 1.0337) 0.1351 | 0.6721 (0.5701, 0.7923) <0.0001 | 0.7300 (0.6180, 0.8624) 0.0003 |
| **p for trend** | 0.9703 (0.9300, 1.0123) 0.1653 | 0.8825 (0.8373, 0.9302) <0.0001 | 0.9070 (0.8605, 0.9561) 0.0004 |
| **Vitamin C** | 0.9996 (0.9990, 1.0002) 0.2372 | 0.9993 (0.9986, 1.0001) 0.1058 | 1.0001 (0.9993, 1.0009) 0.8164 |
| **Vitamin C Quartile** |  |  |  |
| **Q1** | Ref. | Ref. | Ref. |
| **Q2** | 1.0391 (0.9097, 1.1868) 0.5731 | 0.9379 (0.8051, 1.0927) 0.4126 | 0.9867 (0.8456, 1.1514) 0.8653 |
| **Q3** | 1.0228 (0.8958, 1.1678) 0.7392 | 0.8388 (0.7321, 0.9611) 0.0126 | 0.9291 (0.8149, 1.0592) 0.2737 |
| **Q4** | 0.9103 (0.8000, 1.0359) 0.1563 | 0.7842 (0.6755, 0.9104) 0.0018 | 0.9169 (0.7849, 1.0711) 0.2762 |
| **p for trend** | 0.9724 (0.9323, 1.0143) 0.1953 | 0.9192 (0.8776, 0.9627) 0.0005 | 0.9682 (0.9228, 1.0159) 0.1906 |
| **Vitamin E** | 0.9715 (0.9600, 0.9831) <0.0001 | 0.9808 (0.9684, 0.9933) 0.0033 | 0.9855 (0.9732, 0.9980) 0.0244 |
| **Vitamin E Quartile** |  |  |  |
| **Q1** | Ref. | Ref. | Ref. |
| **Q2** | 0.8563 (0.7524, 0.9747) 0.0203 | 0.8670 (0.7520, 0.9996) 0.0515 | 0.8934 (0.7719, 1.0340) 0.1335 |
| **Q3** | 0.7655 (0.6574, 0.8915) 0.0008 | 0.8066 (0.6789, 0.9584) 0.0159 | 0.8276 (0.6962, 0.9838) 0.0340 |
| **Q4** | 0.6612 (0.5710, 0.7656) <0.0001 | 0.7424 (0.6279, 0.8779) 0.0007 | 0.7955 (0.6692, 0.9457) 0.0108 |
| **p for trend** | 0.8735 (0.8329, 0.9160) <0.0001 | 0.9084 (0.8604, 0.9590) 0.0007 | 0.9270 (0.8763, 0.9806) 0.0094 |
| **Zinc** | 0.9831 (0.9677, 0.9989) 0.0377 | 0.9948 (0.9819, 1.0079) 0.4335 | 0.9952 (0.9837, 1.0069) 0.4228 |
| **Zinc Quartile** |  |  |  |
| **Q1** | Ref. | Ref. | Ref. |
| **Q2** | 0.8440 (0.7306, 0.9750) 0.0228 | 0.8961 (0.7630, 1.0525) 0.1837 | 0.8916 (0.7497, 1.0604) 0.1973 |
| **Q3** | 0.8007 (0.6940, 0.9238) 0.0028 | 0.9208 (0.7841, 1.0815) 0.3166 | 0.9159 (0.7716, 1.0872) 0.3172 |
| **Q4** | 0.6553 (0.5612, 0.7653) <0.0001 | 0.7887 (0.6632, 0.9379) 0.0082 | 0.7922 (0.6639, 0.9452) 0.0110 |
| **p for trend** | 0.8767 (0.8336, 0.9220) <0.0001 | 0.9344 (0.8835, 0.9882) 0.0190 | 0.9356 (0.8826, 0.9918) 0.0271 |
| **Selenium** | 0.9958 (0.9945, 0.9972) <0.0001 | 0.9984 (0.9970, 0.9998) 0.0226 | 0.9984 (0.9970, 0.9997) 0.0224 |
| **Selenium Quartile** |  |  |  |
| **Q1** | Ref. | Ref. | Ref. |
| **Q2** | 0.8910 (0.7738, 1.0260) 0.1112 | 0.9538 (0.8145, 1.1168) 0.5578 | 0.9667 (0.8223, 1.1365) 0.6825 |
| **Q3** | 0.7317 (0.6195, 0.8643) 0.0003 | 0.8444 (0.7012, 1.0167) 0.0766 | 0.8301 (0.6811, 1.0116) 0.0675 |
| **Q4** | 0.5795 (0.4912, 0.6836) <0.0001 | 0.8068 (0.6730, 0.9672) 0.0219 | 0.7990 (0.6673, 0.9566) 0.0161 |
| **p for trend** | 0.8340 (0.7899, 0.8806) <0.0001 | 0.9261 (0.8711, 0.9846) 0.0153 | 0.9206 (0.8651, 0.9797) 0.0103 |
| **Carotenoid** | 1.0000 (1.0000, 1.0000) 0.0017 | 1.0000 (1.0000, 1.0000) 0.1309 | 1.0000 (1.0000, 1.0000) 0.5832 |
| **Carotenoid Quartile** |  |  |  |
| **Q1** | Ref. | Ref. | Ref. |
| **Q2** | 0.8856 (0.7724, 1.0154) 0.0841 | 0.9384 (0.8071, 1.0910) 0.4096 | 0.9538 (0.8184, 1.1117) 0.5466 |
| **Q3** | 0.8191 (0.6961, 0.9638) 0.0175 | 0.9409 (0.7846, 1.1284) 0.5125 | 0.9913 (0.8282, 1.1865) 0.9240 |
| **Q4** | 0.7685 (0.6653, 0.8878) 0.0005 | 0.8532 (0.7224, 1.0076) 0.0637 | 0.9137 (0.7731, 1.0800) 0.2923 |
| **p for trend** | 0.9167 (0.8740, 0.9615) 0.0005 | 0.9536 (0.9038, 1.0062) 0.0854 | 0.9769 (0.9258, 1.0308) 0.3957 |

Crude models did not adjust for any covariates; model 1 adjusted for age, sex, race/ethnicity, education, PIR, and marital status; and model 2 additionally adjusted for smoking, alcohol consumption, physical activity, BMI, diabetes, and hypertension based on model 1.

**Table S3**. Association of CDAI with prevalence of specific CVD types in dyslipidemia populations.

|  | **Crude Model**  **OR (95%CI) P-value** | **Model 1**  **OR (95%CI) P-value** | **Model 2**  **OR (95%CI) P-value** |
| --- | --- | --- | --- |
| **CHD** |  |  |  |
| **CDAI** | 0.965 (0.945, 0.984) 0.0007 | 0.978 (0.957, 0.987) 0.0026 | 0.978 (0.957, 1.000) 0.0492 |
| **CDAI quartile** |  |  |  |
| Q1 | Ref. | Ref. | Ref. |
| Q2 | 0.977 (0.805, 1.187) 0.8180 | 0.939 (0.760, 1.160) 0.5594 | 0.938 (0.759, 1.159) 0.5516 |
| Q3 | 0.818 (0.682, 0.981) 0.0324 | 0.829 (0.683, 1.005) 0.0580 | 0.827 (0.682, 1.002) 0.0549 |
| Q4 | 0.646 (0.530, 0.787) <0.0001 | 0.741 (0.595, 0.924) 0.0087 | 0.738 (0.591, 0.922) 0.0084 |
| P for trend | <0.0001 | 0.0038 | 0.0037 |
| **CHF** |  |  |  |
| **CDAI** | 0.927 (0.901, 0.954) <0.0001 | 0.963 (0.938, 0.989) 0.0065 | 0.965 (0.940, 0.991) 0.0096 |
| **CDAI quartile** |  |  |  |
| Q1 | Ref. | Ref. | Ref. |
| Q2 | 0.908 (0.736, 1.120) 0.3678 | 1.035 (0.835, 1.283) 0.7538 | 1.039 (0.839, 1.286) 0.7272 |
| Q3 | 0.696 (0.563, 0.861) 0.0011 | 0.903 (0.725, 1.124) 0.3613 | 0.912 (0.732, 1.135) 0.4104 |
| Q4 | 0.486 (0.370, 0.640) <0.0001 | 0.726 (0.547, 0.964) 0.0285 | 0.741 (0.557, 0.987) 0.0425 |
| P for trend | <0.0001 | 0.0151 | 0.0250 |
| **Heart Attack** |  |  |  |
| **CDAI** | 0.950 (0.926, 0.975) 0.0001 | 0.973 (0.948, 0.999) 0.0413 | 0.974 (0.949, 0.999) 0.0447 |
| **CDAI quartile** |  |  |  |
| Q1 | Ref. | Ref. | Ref. |
| Q2 | 0.882 (0.738, 1.055) 0.1717 | 0.913 (0.750, 1.112) 0.3679 | 0.913 (0.749, 1.112) 0.3656 |
| Q3 | 0.744 (0.596, 0.929) 0.0101 | 0.844 (0.659, 1.081) 0.1815 | 0.845 (0.659, 1.083) 0.1852 |
| Q4 | 0.560 (0.440, 0.713) <0.0001 | 0.717 (0.551, 0.933) 0.0146 | 0.721 (0.554, 0.939) 0.0166 |
| P for trend | <0.0001 | 0.0170 | 0.0192 |
| **Stroke** |  |  |  |
| **CDAI** | 0.924 (0.903, 0.946) <0.0001 | 0.955 (0.932, 0.979) 0.0003 | 0.957 (0.934, 0.981) 0.0008 |
| **CDAI quartile** |  |  |  |
| Q1 | Ref. | Ref. | Ref. |
| Q2 | 0.762 (0.618, 0.939) 0.0118 | 0.846 (0.676, 1.059) 0.1460 | 0.848 (0.678, 1.061) 0.1527 |
| Q3 | 0.581 (0.473, 0.713) <0.0001 | 0.717 (0.578, 0.889) 0.0029 | 0.724 (0.583, 0.899) 0.0042 |
| Q4 | 0.451 (0.369, 0.551) <0.0001 | 0.623 (0.504, 0.771) <0.0001 | 0.637 (0.512, 0.792) 0.0001 |
| P for trend | <0.0001 | <0.0001 | <0.0001 |
| **Angina** |  |  |  |
| **CDAI** | 0.947 (0.919, 0.976) 0.0006 | 0.965 (0.936, 0.995) 0.0246 | 0.967 (0.937, 0.997) 0.0349 |
| **CDAI quartile** |  |  |  |
| Q1 | Ref. | Ref. | Ref. |
| Q2 | 0.986 (0.767, 1.268) 0.9131 | 1.022 (0.789, 1.324) 0.8694 | 1.025 (0.791, 1.328) 0.8510 |
| Q3 | 0.817 (0.616, 1.083) 0.1621 | 0.908 (0.682, 1.208) 0.5077 | 0.916 (0.688, 1.221) 0.5525 |
| Q4 | 0.622 (0.449, 0.860) 0.0048 | 0.765 (0.546, 0.872) 0.0079 | 0.778 (0.552, 0.997) 0.0084 |
| P for trend | 0.0017 | 0.0024 | 0.0093 |

Crude models did not adjust for any covariates; model 1 adjusted for age, sex, race/ethnicity, education, PIR, and marital status; and model 2 additionally adjusted for smoking, alcohol consumption, physical activity, BMI, diabetes, and hypertension based on model 1.

**Table S4**. Threshold effect analysis of the association of CDAI with CHD and heart attack in dyslipidemia.

|  | CDAI ≤ 0 | CDAI >0 | P-interaction |
| --- | --- | --- | --- |
|  | **OR (95%CI) P-value** | **OR (95%CI) P-value** |  |
| **CHD** |  |  |  |
| CDAI | 0.939 (0.877, 0.985) 0.0023 | 1.003 (0.978, 1.029) 0.7979 | 0.0509 |
| **Heart Attack** |  |  |  |
| CDAI | 0.940 (0.878, 0.997) 0.0473 | 0.999 (0.968, 1.032) 0.9606 | 0.1226 |

**Table S5**. Association of CDAI components with CVD mortality in dyslipidemia populations.

|  | **Crude model**  **HR (95%CI) P-value** | **Model 1**  **HR (95%CI) P-value** | **Model 2**  **HR (95%CI) P-value** |
| --- | --- | --- | --- |
| **Vitamin A** | 1.0000 (1.0000, 1.0001) 0.3980 | 1.0000 (0.9999, 1.0001) 0.4328 | 1.0000 (0.9999, 1.0001) 0.7663 |
| **Vitamin A Quartile** |  |  |  |
| **Q1** | 1.0 | 1.0 | 1.0 |
| **Q2** | 1.1270 (0.9590, 1.3244) 0.1465 | 1.1637 (0.9893, 1.3688) 0.0671 | 1.0628 (0.9031, 1.2507) 0.4632 |
| **Q3** | 1.2054 (1.0278, 1.4136) 0.0215 | 1.2414 (1.0561, 1.4590) 0.0087 | 1.0682 (0.9077, 1.2570) 0.4270 |
| **Q4** | 1.0925 (0.9292, 1.2845) 0.2840 | 1.1038 (0.9350, 1.3031) 0.2434 | 0.9673 (0.8184, 1.1433) 0.6966 |
| **p for trend** | 1.0332 (0.9829, 1.0861) 0.1998 | 1.0356 (0.9840, 1.0899) 0.1793 | 0.9898 (0.9396, 1.0427) 0.6999 |
| **Vitamin C** | 0.9992 (0.9984, 0.9999) 0.0203 | 0.9999 (0.9993, 1.0006) 0.8190 | 1.0002 (0.9995, 1.0009) 0.5658 |
| **Vitamin C Quartile** |  |  |  |
| **Q1** | 1.0 | 1.0 | 1.0 |
| **Q2** | 0.9799 (0.8326, 1.1533) 0.8069 | 1.1066 (0.9397, 1.3031) 0.2246 | 1.0180 (0.8638, 1.1996) 0.8318 |
| **Q3** | 1.1243 (0.9604, 1.3161) 0.1451 | 1.3523 (1.1538, 1.5850) 0.0001 | 1.1829 (1.0075, 1.3887) 0.0401 |
| **Q4** | 0.9724 (0.8285, 1.1412) 0.7314 | 1.2270 (1.0432, 1.4433) 0.013501 | 1.1998 (1.0181, 1.4140) 0.0296 |
| **p for trend** | 1.0048 (0.9559, 1.0563) 0.8506 | 1.0839 (1.0307, 1.1399) 0.0017 | 1.0724 (1.0181, 1.1295) 0.0083 |
| **Vitamin E** | 0.9421 (0.9275, 0.9569) <0.0001 | 0.9480 (0.9332, 0.9630) <0.0001 | 0.9534 (0.9385, 0.9686) <0.0001 |
| **Vitamin E Quartile** |  |  |  |
| **Q1** | 1.0 | 1.0 | 1.0 |
| **Q2** | 0.8156 (0.7050, 0.9436) 0.0061 | 0.8861 (0.7646, 1.0268) 0.1076 | 0.9134 (0.7879, 1.0588) 0.2293 |
| **Q3** | 0.7376 (0.6335, 0.8588) 0.0088 | 0.7934 (0.6791, 0.9269) 0.0035 | 0.8177 (0.6998, 0.9556) 0.0113 |
| **Q4** | 0.5708 (0.4822, 0.6758) <0.0001 | 0.6122 (0.5138, 0.7294) <0.0001 | 0.6659 (0.5582, 0.7943) 0.0006 |
| **p for trend** | 0.8387 (0.7966, 0.8831) <0.0001 | 0.8592 (0.8146, 0.9063) <0.0001 | 0.8804 (0.8342, 0.9290) 0.0003 |
| **Zinc** | 0.9666 (0.9564, 0.9769) <0.0001 | 0.9595 (0.9489, 0.9703) <0.0001 | 0.9736 (0.9629, 0.9844) 0.0002 |
| **Zinc Quartile** |  |  |  |
| **Q1** | 1.0 | 1.0 | 1.0 |
| **Q2** | 0.9350 (0.8046, 1.0865) 0.3802 | 0.9559 (0.8213, 1.1125) 0.5597 | 0.9648 (0.8284, 1.1236) 0.6447 |
| **Q3** | 0.8014 (0.6865, 0.9356) 0.0050 | 0.7965 (0.6792, 0.9340) 0.0051 | 0.8952 (0.7624, 1.0512) 0.1768 |
| **Q4** | 0.5918 (0.5023, 0.6974) <0.0001 | 0.5324 (0.4476, 0.6332) <0.0001 | 0.6567 (0.5515, 0.7821) 0.0002 |
| **p for trend** | 0.8466 (0.8052, 0.8901) <0.0001 | 0.8201 (0.7778, 0.8647) <0.0001 | 0.8805 (0.8343, 0.9291) 0.0004 |
| **Selenium** | 0.9944 (0.9932, 0.9957) <0.0001 | 0.9938 (0.9925, 0.9951) <0.0001 | 0.9961 (0.9947, 0.9974) <0.0001 |
| **Selenium Quartile** |  |  |  |
| **Q1** | 1.0 | 1.0 | 1.0 |
| **Q2** | 0.8123 (0.7036, 0.9379) 0.0046 | 0.8186 (0.7080, 0.9465) 0.0068 | 0.8740 (0.7556, 1.0110) 0.0697 |
| **Q3** | 0.6844 (0.5877, 0.7969) 0.0001 | 0.6585 (0.5630, 0.7702) <0.0001 | 0.7237 (0.6182, 0.8472) 0.0057 |
| **Q4** | 0.4451 (0.3742, 0.5295) <0.0001 | 0.4028 (0.3355, 0.4837) <0.0001 | 0.5552 (0.4615, 0.6679) <0.0001 |
| **p for trend** | 0.7802 (0.7409, 0.8215) <0.0001 | 0.7565 (0.7162, 0.7990) <0.0001 | 0.8287 (0.7836, 0.8764) <0.0001 |
| **Carotenoid** | 1.0000 (1.0000, 1.0000) 0.0001 | 1.0000 (1.0000, 1.0000) 0.0043 | 1.0000 (1.0000, 1.0000) 0.0074 |
| **Carotenoid Quartile** |  |  |  |
| **Q1** | 1.0 | 1.0 | 1.0 |
| **Q2** | 0.9828 (0.8440, 1.1444) 0.8230 | 1.1128 (0.9548, 1.2969) 0.1712 | 1.1441 (0.9812, 1.3340) 0.0853 |
| **Q3** | 0.8294 (0.7076, 0.9722) 0.0209 | 0.9905 (0.8436, 1.1630) 0.9075 | 1.0432 (0.8878, 1.2259) 0.6073 |
| **Q4** | 0.7733 (0.6593, 0.9070) 0.0015 | 0.8969 (0.7630, 1.0542) 0.1869 | 0.9248 (0.7863, 1.0875) 0.3443 |
| **p for trend** | 0.9112 (0.8666, 0.9579) 0.0002 | 0.9595 (0.9126, 1.0088) 0.1057 | 0.9709 (0.9234, 1.0208) 0.2482 |

Crude models did not adjust for any covariates; model 1 adjusted for age, sex, race/ethnicity, education, PIR, and marital status; and model 2 additionally adjusted for smoking, alcohol consumption, physical activity, BMI, diabetes, and hypertension based on model 1.

**Table S6**. Association of CDAI with CVD mortality in dyslipidemia populations excluding those with a follow-up length of two years.

|  | **Crude Model**  **HR (95%CI)** | **p** | **Model 1**  **HR (95%CI)** | **p** | **Model 2**  **HR (95%CI)** | **p** |
| --- | --- | --- | --- | --- | --- | --- |
| **CDAI** | 0.937(0.919,0.956) | <0.0001 | 0.956(0.938,0.975) | <0.0001 | 0.960(0.940,0.980) | <0.001 |
| **CDAI quartile** |  |  |  |  |  |  |
| Q1 | ref | ref | ref | ref | ref | ref |
| Q2 | 1.217(0.991,1.494) | 0.062 | 1.344(1.099,1.644) | 0.004 | 1.292(1.048,1.592) | 0.017 |
| Q3 | 0.851(0.687,1.054) | 0.14 | 1.018(0.825,1.257) | 0.865 | 1.024(0.833,1.258) | 0.824 |
| Q4 | 0.614(0.491,0.768) | <0.0001 | 0.756(0.601,0.950) | 0.016 | 0.785(0.627,0.984) | 0.036 |
| P for trend | <0.0001 | | <0.001 | | 0.004 | |

Crude models did not adjust for any covariates; model 1 adjusted for age, sex, race/ethnicity, education, PIR, and marital status; and model 2 additionally adjusted for smoking, alcohol consumption, physical activity, BMI, diabetes, and hypertension based on model 1.

**Table S7**. Baseline analysis of HEI-2015 levels according to CDAI quartiles, NHANES 2001-2018.

|  | **Total** | **Q1** | **Q2** | **Q3** | **Q4** | **P-value** |
| --- | --- | --- | --- | --- | --- | --- |
| **HEI-2015** | 53.108±0.193 | 47.736±0.269 | 51.760±0.252 | 54.317±0.236 | 57.295±0.270 | <0.0001 |

HE-2015 were expressed as mean ± standard error and tested by weighted analysis of variance (ANOVA).

**Table S8**. Association of CDAI with CVD/ASCVD and CVD mortality in people with dyslipidemia (additional adjustment for HEI-2015).

|  | CVD  Model2 +HEI-2015  OR (95%CI) | P-value | ASCVD  Model2 +HEI-2015  OR (95%CI) | P-value | CVD mortality  Model2 +HEI-2015  HR (95%CI) | P-value |
| --- | --- | --- | --- | --- | --- | --- |
| CDAI | 0.981(0.965,0.999) | 0.035 | 0.979(0.962,0.996) | 0.018 | 0.949(0.929,0.969) | <0.0001 |
| CDAI quartile |  |  |  |  |  |  |
| Q1 | ref | ref | ref | ref | ref | ref |
| Q2 | 0.925(0.793,1.079) | 0.318 | 0.909(0.770,1.073) | 0.257 | 1.121(0.917,1.372) | 0.265 |
| Q3 | 0.891(0.747,1.062) | 0.196 | 0.868(0.719,1.049) | 0.141 | 0.907(0.760,1.082) | 0.279 |
| Q4 | 0.799(0.667,0.958) | 0.016 | 0.773(0.643,0.929) | 0.007 | 0.680(0.549,0.842) | <0.001 |
| P for trend | 0.021 | | 0.009 | | <0.0001 | |

Adjusted for age, sex, race/ethnicity, education, PIR, marital status, smoking, alcohol consumption, physical activity, BMI, diabetes, hypertension, and HEI-2015.

**Table S9**. Stratified analysis according to BMI (<30 and ≥30).

|  | **OR (95%CI)** | **P value** | **P for interaction** |
| --- | --- | --- | --- |
| CVD |  |  |  |
| BMI |  |  | 0.807 |
| <30 | 0.975(0.960,0.991) | 0.002 |  |
| >=30 | 0.979(0.963,0.996) | 0.015 |  |
| ASCVD |  |  |  |
| BMI |  |  | 0.79 |
| <30 | 0.975(0.959,0.990) | 0.002 |  |
| >=30 | 0.98(0.962,0.997) | 0.022 |  |
|  | **HR (95%CI)** | **P value** | **P for interaction** |
| CVD mortality |  |  |  |
| BMI |  |  | 0.007 |
| <30 | 0.971(0.952,0.990) | 0.004 |  |
| >=30 | 0.954(0.927,0.981) | 0.001 |  |

**Table S10**. Association of CDAI with prevalence of CVD/ASCVD in dyslipidemic populations before and after adjustment for BMI.

|  | Model 2  OR (95%CI) P-value | Model 2 (before adjustment for BMI)  OR (95%CI) P-value |
| --- | --- | --- |
| CVD |  |  |
| CDAI | 0.979 (0.964, 0.995) 0.0132 | 0.979(0.964,0.996)  0.0131 |
| CDAI quartile |  |  |
| Q1 | Ref. | Ref. |
| Q2 | 0.906 (0.779, 1.054) 0.2035 | 0.917(0.787,1.069)  2650 |
| Q3 | 0.874 (0.739, 1.033) 0.1177 | 0.878(0.741,1.041)  0.1329 |
| Q4 | 0.780 (0.656, 0.926) 0.0054 | 0.783(0.659,0.931)  0.0058 |
| P for trend | 0.0081 | 0.0080 |
| ASCVD |  |  |
| CDAI | 0.977 (0.961, 0.993) 0.0057 | 0.977(0.961,0.993)  0.0058 |
| CDAI quartile |  |  |
| Q1 | Ref. | ref |
| Q2 | 0.892 (0.758, 1.050) 0.1715 | 0.901(0.764,1.062)  0.2110 |
| Q3 | 0.852 (0.712, 1.021) 0.0851 | 0.855(0.712,1.027)  0.0917 |
| Q4 | 0.754 (0.634, 0.898) 0.0020 | 0.757(0.635,0.902)  0.0020 |
| P for trend | 0.0031 | 0.0032 |

**Table S11**. Association of CDAI with CVD mortality in dyslipidemic populations before and after adjustment for BMI.

|  | Model 2 HR (95%CI) | P-value | Model 2 ( (before adjustment for BMI) HR (95%CI) | P-value |
| --- | --- | --- | --- | --- |
| CDAI | 0.957(0.939,0.976) | <0.0001 | 0.956(0.937,0.975) | <0.0001 |
| CDAI quartile |  |  |  |  |
| Q1 | ref | ref | ref | ref |
| Q2 | 1.180(0.962,1.448) | 0.112 | 1.156(0.945,1.415) | 0.158 |
| Q3 | 0.959(0.802,1.147) | 0.648 | 0.949(0.794,1.134) | 0.564 |
| Q4 | 0.745(0.603,0.921) | 0.006 | 0.728(0.589,0.900) | 0.003 |
| P for trend | <0.001 | | <0.001 | |

**Table S12**. Association of CDAI with CVD prevalence in people without dyslipidemia.

| CVD | Crude Model  OR (95%CI) | p | Model 1  OR (95%CI) | p | Model 2  OR (95%CI) | p |
| --- | --- | --- | --- | --- | --- | --- |
| CDAI | 0.954(0.935,0.973) | <0.0001 | 0.976(0.958,0.995) | 0.016 | 0.984(0.965,1.004) | 0.108 |
| CDAI quartile |  |  |  |  |  |  |
| Q1 | ref | ref | ref | ref | ref | ref |
| Q2 | 0.852(0.749,0.969) | 0.015 | 0.878(0.759,1.015) | 0.079 | 0.876(0.754,1.017) | 0.081 |
| Q3 | 0.725(0.628,0.837) | <0.0001 | 0.823(0.702,0.964) | 0.016 | 0.864(0.736,1.013) | 0.071 |
| Q4 | 0.601(0.503,0.717) | <0.0001 | 0.758(0.625,0.920) | 0.005 | 0.792(0.647,0.969) | 0.024 |
| P for trend | <0.0001 | | 0.005 | | 0.032 | |

Crude models did not adjust for any covariates; model 1 adjusted for age, sex, race/ethnicity, education, PIR, and marital status; and model 2 additionally adjusted for smoking, alcohol consumption, physical activity, BMI, diabetes, and hypertension based on model 1.

**Table S13**. Association of CDAI with ASCVD prevalence in people without dyslipidemia.

| ASCVD | Crude Model  OR (95%CI) | p | Model 1  OR (95%CI) | p | Model 2  OR (95%CI) | p |
| --- | --- | --- | --- | --- | --- | --- |
| CDAI | 0.947(0.928,0.966) | <0.0001 | 0.969(0.949,0.990) | 0.004 | 0.970(0.942,1.000) | 0.051 |
| CDAI quartile |  |  |  |  |  |  |
| Q1 | ref | ref | ref | ref | ref | ref |
| Q2 | 0.837(0.730,0.959) | 0.011 | 0.855(0.732,0.999) | 0.049 | 0.850(0.717,1.007) | 0.060 |
| Q3 | 0.711(0.609,0.829) | <0.0001 | 0.799(0.674,0.948) | 0.011 | 0.826(0.678,1.006) | 0.057 |
| Q4 | 0.570(0.474,0.684) | <0.0001 | 0.714(0.583,0.874) | 0.001 | 0.718(0.556,0.928) | 0.012 |
| P for trend | <0.0001 | | 0.002 | | 0.022 | |

Crude models did not adjust for any covariates; model 1 adjusted for age, sex, race/ethnicity, education, PIR, and marital status; and model 2 additionally adjusted for smoking, alcohol consumption, physical activity, BMI, diabetes, and hypertension based on model 1.

**Table S14**. Association of CDAI with CVD mortality in people without dyslipidemia.

| CVD mortality | Crude Model HR (95%CI) | p | Model 1 HR (95%CI) | p | Model 2 HR (95%CI) | p |
| --- | --- | --- | --- | --- | --- | --- |
| CDAI | 0.936(0.920,0.954) | <0.0001 | 0.994(0.969,1.019) | 0.637 | 1.001(0.977,1.027) | 0.911 |
| CDAI quartile |  |  |  |  |  |  |
| Q1 | ref | ref | ref | ref | ref | ref |
| Q2 | 0.985(0.826,1.173) | 0.863 | 1.084(0.913,1.288) | 0.358 | 1.129(0.948,1.345) | 0.175 |
| Q3 | 0.706(0.596,0.837) | <0.0001 | 0.951(0.794,1.140) | 0.586 | 1.014(0.843,1.220) | 0.882 |
| Q4 | 0.544(0.441,0.672) | <0.0001 | 1.044(0.821,1.328) | 0.727 | 1.123(0.888,1.421) | 0.333 |
| P for trend | <0.0001 | | 0.9012 | | 0.5680 | |

Crude models did not adjust for any covariates; model 1 adjusted for age, sex, race/ethnicity, education, PIR, and marital status; and model 2 additionally adjusted for smoking, alcohol consumption, physical activity, BMI, diabetes, and hypertension based on model 1.

**Table S15.** Baseline analysis of dietary energy and nutrient intakes levels according to CDAI quartiles, NHANES 2001-2018.

| **Characteristics** | **Total (n=23126)** | **Q1 (n=5787)** | **Q2 (n=5776)** | **Q3 (n=5781)** | **Q4 (n=5782)** | **P value** |
| --- | --- | --- | --- | --- | --- | --- |
| **CDAI** | 0.782 ± 0.058 | -3.712 ± 0.022 | -1.194 ± 0.011 | 1.013 ± 0.012 | 5.755 ± 0.082 | <0.0001 |
| **Vitamin A, mcg** | 640.252±7.533 | 279.072± 3.260 | 478.227± 4.447 | 643.880± 4.958 | 1056.653±20.133 | < 0.0001 |
| **Vitamin C, mg** | 81.758±0.948 | 35.265±0.609 | 58.311±0.751 | 84.770±1.045 | 135.195±1.891 | < 0.0001 |
| **Vitamin E, as alpha tocopherol mg** | 8.018±0.071 | 3.902±0.033 | 6.098±0.042 | 8.147±0.055 | 12.743±0.143 | < 0.0001 |
| **Zinc, mg** | 11.717±0.080 | 6.638±0.050 | 9.784±0.059 | 12.123±0.081 | 16.937±0.202 | < 0.0001 |
| **Selenium, mcg** | 111.741±0.577 | 68.049±0.449 | 96.896±0.521 | 117.396±0.565 | 153.079±1.208 | < 0.0001 |
| **Carotenoid, mcg** | 9849.187±143.744 | 3304.637± 52.948 | 6275.798± 85.022 | 9427.564±110.101 | 18405.631±302.168 | < 0.0001 |
| **Energy intake, kcal/day** | 2156.734±8.908 | 1471.708±10.896 | 1936.652±14.083 | 2279.045±11.793 | 2762.264±19.754 | < 0.0001 |
| **Fat intake, g/day** | 83.569±0.430 | 53.245±0.525 | 74.400±0.645 | 89.009±0.691 | 109.852±1.007 | < 0.0001 |

**Table S16**. Association of CDAI with CVD/ASCVD in people with dyslipidemia (additional adjustment for energy intake and dietary fat).

|  | CVD Model  OR (95%CI) P-value | ASCVD Model  OR (95%CI) P-value |
| --- | --- | --- |
| CDAI | 0.979(0.964,0.996)  0.013 | 0.977(0.961,0.993)  0.006 |
| CDAI quartile |  |  |
| Q1 | ref | ref |
| Q2 | 0.917(0.787,1.069)  0.2651 | 0.901(0.764,1.062)  0.211 |
| Q3 | 0.878(0.741,1.041)  0.1334 | 0.855(0.712,1.027)  0.0930 |
| Q4 | 0.783(0.659,0.931)  0.0062 | 0.757(0.635,0.902)  0.0023 |
| P for trend | 0.0086 | 0.0037 |

Adjust for age, sex, race, PIR, marital status, educational level, smoking, drinking, physical activity, diabetes, hypertension, BMI, energy intake and dietary fat

**Table S17**. Association of CDAI with CVD mortality in people with dyslipidemia (additional adjustment for energy intake and dietary fat).

| CVD-death | Model  HR (95%CI) | p |
| --- | --- | --- |
| CDAI | 0.956(0.937,0.975) | <0.0001 |
| CDAI quartile |  |  |
| Q1 | ref | ref |
| Q2 | 1.156(0.945,1.415) | 0.158 |
| Q3 | 0.949(0.794,1.134) | 0.564 |
| Q4 | 0.728(0.589,0.900) | 0.003 |
| P for trend | <0.001 | |

Adjust for age, sex, race, PIR, marital status, educational level, smoking, drinking, physical activity, diabetes, hypertension, BMI, energy intake and dietary fat

Figure S1. Visualizing Causal and Confounding Paths in the Link Between CDAI and CVD


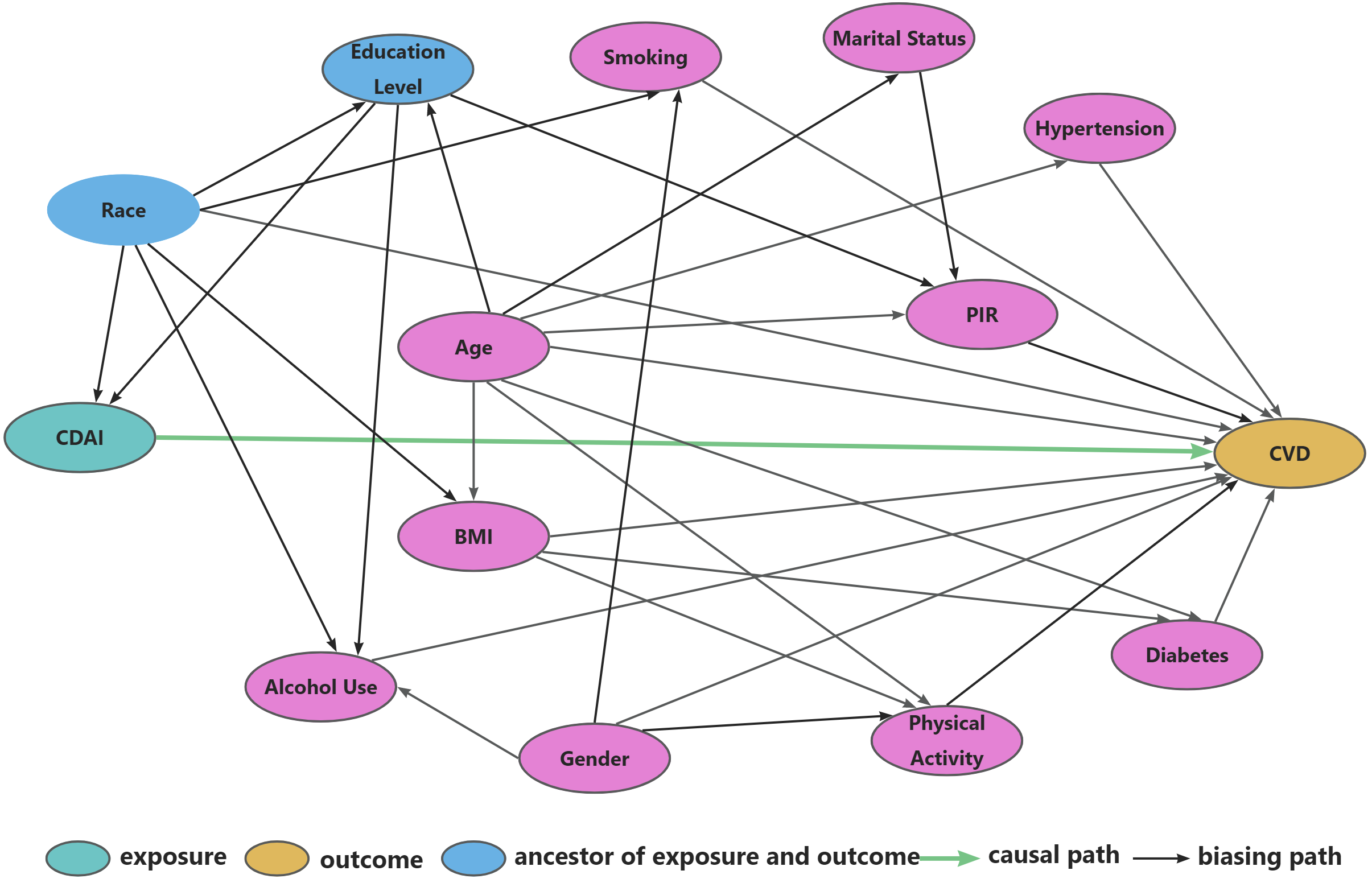

Supplement: Supplementary file 1 [file Data_Sheet_1.docx]
